# Supplementary material for: Progress in the molecular phylogeny of Cotesia acuminata and C. melitaearum cryptic species complexes
Source: PLoS One. 2026 May 27;21(5):e0348973. doi: 10.1371/journal.pone.0348973 (PMC13215553; doi:10.1371/journal.pone.0348973)
Supplement: S1 Data — Table A. Ten-gene partitioning. Ten-gene dataset partitions determined by partitioning algorithm, and best fit model as determined by ModelFinder using BIC. Table B. Nuclear partitioning. Nuclear gene dataset partitions determined by partitioning algorithm, and best fit model determined by ModelFinder using BIC. Table C. Mitochondrial partitioning. Mitochondrial-gene dataset partitions determined by partitioning algorithm, and best fit model determined by ModelFinder using BIC. Table D. Substitution models. Substitution models used for individual genes, determined by ModelFinder, and selected based on BIC. Table E. Primer pairs. Characteristics of the additional primer pairs created for this study. Includes the marker targeted (gene), primer name, primer sequence, amplicon size. (DOCX) [file pone.0348973.s001.docx]

Table A.

| **Partition ID** | **Genes** | **Model** | **BIC** |
| --- | --- | --- | --- |
| 1 | *16s* | K3Pu+F+G4 | 3902.492 |
| 2 | *18s, LW-Rh, Elob* | K3Pu+F+R2 | 4787.116 |
| 3 | *28s, EF1a1* | TPM2u+F+I+R2 | 6547.643 |
| 4 | *COI, INX* | TIM+F+G4 | 8279.967 |
| 5 | *ITS2, SLD5* | TPM2u+F+I+R2 | 6314.743 |

Table B.

| **Partition ID** | **Genes** | **Model** | **BIC** |
| --- | --- | --- | --- |
| 1 | *18s, 28s, Ef1a1, LW-Rh, Elob* | TPM2u+F+R2 | 11228.316 |
| 2 | *INX, ITS2, SLD5* | TPM2u+F+I+R2 | 8211.923 |

Table C.

| **Partition ID** | **Genes** | **Model** | **BIC** |
| --- | --- | --- | --- |
| 1 | *16s* | K3Pu+F+G4 | 3423.034 |
| 2 | *COI* | TIM+F+G4 | 5850.518 |

Table D.

| **Gene** | **Model** | **BIC** |
| --- | --- | --- |
| 16s | K3Pu+F+G4 | 4443.699 |
| 18s | K2P+G4 | 2197.826 |
| 28s | HKY+F+R2 | 4634.309 |
| COI | TIM+F+G4 | 6982.191 |
| EF1A1 | TIM2e+I | 3078.133 |
| INX | HKY+F+G4 | 2519.785 |
| ITS2 | F81+F+G4 | 4770.091 |
| LW-Rh | HKY+F+G4 | 1941.431 |
| SLD5 | K2P+G4 | 2524.745 |
| Elob | HKY+F+G4 | 2057.275 |

Table E.

| **Gene** | **Primer name** | **Sequence (5'-3')** | **Amplicon size** |
| --- | --- | --- | --- |
| *DAP1* | DAP-F | 5`-CAAAACACCACGGGACGAAC | 132 |
|  | DAP-R | 5`-TCTGGAGGAAAATCGGCGTT |  |
| *PSMB* | PSMB-F | CGCCTGAGATTCCGGCTAAA | 368 |
|  | PSMB-R | 5`-TGTACGATAGCACCCCAACC |  |
| *SBDS* | SBDS-F | 5`-TCGGCAATGACAACCAGACA | 305 |
|  | SBDS-R | 5`-GACCCTAACTCGCATCCTGG |  |
| *NUTF2* | NUTF2-F | 5`-GCTCAATAGCCTGGGTTTCCA | 256 |
|  | NUTF2-R | 5`-CAGTGGCTTCAGCACAAACG |  |
| *CPSF5* | CPSF5-F | 5`-GCGTAGGAGTGTCGAAGGAG | 375 |
|  | CPSF5-R | 5`-AGTTCAAACAATGGCGCAGC |  |
| *HIGD2A* | HIGD2A-F | 5`-TCCTTTGGTGCCGATAGGTG | 128 |
|  | HIGD2A-R | 5`-TGAAACCCTGAGCAGATACCC |  |
| *AIMP1* | AIMP1-F | 5`-AGCTCTGGGATGACGTTGAC | 329 |
|  | AIMP1-R | 5`-TTTTGGCGAGTTTTGGTGCG |  |
| *ATG3* | ATG3-F | 5`-ATGGGCAACTGGAGACGAAG | 724 |
|  | ATG3-R | 5`-CGGAATCACCGACTGGACAA |  |
| *INTS2* | INTS2-F | 5`-GGTACAGAAGTCGGTGAGGC | 309 |
|  | INTS2-R | 5`-CAGCAGCGGCGAGAATAAAC |  |
| *Taz* | Taz-F | 5`-TTCGCAAGAAAACGACGCAC | 371 |
|  | Taz-R | 5`-TCATGTGCTGCTAACGACCA |  |
| *B4GALT7* | B4GALT7-F | 5`-CAGTTTGCTCCGCACATGAAA | 357 |
|  | B4GALT7-R | 5`-ATCTTCTAAGCCCCAACCCC |  |
| *SMD2* | SMD2-F | 5`-GGACCACTTTCAGTACTTACCCA | 273 |
|  | SMD2-R | 5`-GGCGGTTGCAAGTGGATTTC |  |
| *STX6* | STX6-F | 5`-CGTGACAGAACTGCGAGACA | 388 |
|  | STX6-R | 5`-ATGCAGTTGTAGGAGCAGGA |  |
