# Supplementary material for: Progress in the molecular phylogeny of Cotesia acuminata and C. melitaearum cryptic species complexes
Source: PLoS One. 2026 May 27;21(5):e0348973. doi: 10.1371/journal.pone.0348973 (PMC13215553; doi:10.1371/journal.pone.0348973)
Supplement: Attachment — Submitted filename: PONE-D-26-02522_PJ.pdf [file pone.0348973.s020.pdf]

PLOS One

Progress in the molecular phylogeny of Cotesiaacuminata and C. melitaearum cryptic species complexes

--Manuscript Draft--

**Manuscript Number:**	PONE-D-26-02522
**Article Type:**	Research Article
**Full Title:**	Progress in the molecular phylogeny of Cotesiaacuminata and C. melitaearum cryptic species complexes
**Short Title:**	Progress in the molecular phylogeny of Cotesia acuminata and C. melitaearum cryptic species complexes
**Corresponding Author:**	Julian Schach<br>University of Helsinki: Helsingin Yliopisto<br>Helsinki, FINLAND
**Keywords:**	Parasitoids, Maximum likelihood, Melitaeini, Systematics, Braconidae
**Abstract:**	Cryptic species present major challenges for biodiversity and evolutionary research due to the morphological similarity and frequent lack of available genetic and ecological data. In parasitoids wasps, this is especially true for lineages with limited sampling and unclear species boundaries. Here we reconstruct the phylogeny of Cotesia wasps parasitizing Melitaeini butterflies, including two cryptic species complexes (Cotesia acuminata agg. and C. melitaearum agg.). Using a ten-gene dataset from samples collected in Europe, Asia, and North America, we inferred relationships among 22 Cotesia species using maximum likelihood. We also included non-Melitaeini associated Cotesia to assess whether the parasitoids of Melitaeini form a monophyletic group. Our analyses yielded a highly supported phylogeny, revealing four major clades, three of which included the Melitaeini associated species. This confirms that the Cotesia parasitizing Melitaeini butterflies are polyphyletic, resulting from independent host shifts across the genus. Each clade is further subdivided into subclades corresponding to the different cryptic species complexes, clarifying previously unresolved relationships. These results provide a robust framework for future studies on the evolution, ecology, and host use dynamics of Cotesia wasps and highlight the utility of multi-locus data for resolving phylogenies in morphologically cryptic taxa.
**Order of Authors:**	Julian Schach
	Federica Valerio
	Anne Duplouy
**Additional Information:**	
**Question**	**Response**
**Financial Disclosure**<br><br>Enter a financial disclosure statement that describes the sources of funding for the work included in this submission. Review the submission guidelines for detailed requirements. View published research articles from *PLOS ONE* for specific examples.<br><br>This statement is required for submission and **will appear in the published article** if the submission is accepted. Please make	Yes

sure it is accurate.<br><br>**Funded studies**<br>Enter a statement with the following details:<br>• Initials of the authors who received each award<br>• Grant numbers awarded to each author<br>• The full name of each funder<br>• URL of each funder website<br>• Did the sponsors or funders play any role in the study design, data collection and analysis, decision to publish, or preparation of the manuscript?<br><br>Did you receive funding for this work?	
Please add funding details.<br>as follow-up to "**Financial Disclosure**<br><br>Enter a financial disclosure statement that describes the sources of funding for the work included in this submission. Review the submission guidelines for detailed requirements. View published research articles from *PLOS ONE* for specific examples.<br><br>This statement is required for submission and **will appear in the published article** if the submission is accepted. Please make sure it is accurate.<br><br>**Funded studies**<br>Enter a statement with the following details:<br>• Initials of the authors who received each award<br>• Grant numbers awarded to each author<br>• The full name of each funder<br>• URL of each funder website<br>• Did the sponsors or funders play any role in the study design, data collection and analysis, decision to publish, or preparation of the manuscript?<br><br>Did you receive funding for this work?"	The work was funded by the Research Council of Finland to Dr. Anne Duplouy grant number 328944
Please select the country of your main research funder (please select carefully	FINLAND - FI

as in some cases this is used in fee calculation).<br>as follow-up to "**Financial Disclosure**<br><br>Enter a financial disclosure statement that describes the sources of funding for the work included in this submission. Review the submission guidelines for detailed requirements. View published research articles from *PLOS ONE* for specific examples.<br><br>This statement is required for submission and **will appear in the published article** if the submission is accepted. Please make sure it is accurate.<br><br>**Funded studies**<br>Enter a statement with the following details:<br>• Initials of the authors who received each award<br>• Grant numbers awarded to each author<br>• The full name of each funder<br>• URL of each funder website<br>• Did the sponsors or funders play any role in the study design, data collection and analysis, decision to publish, or preparation of the manuscript?<br><br>Did you receive funding for this work?"	
**Competing Interests**<br><br>Use the instructions below to enter a competing interest statement for this submission. On behalf of all authors, disclose any competing interests that could be perceived to bias this work—acknowledging all financial support and any other relevant financial or non-financial competing interests.<br><br>This statement is required for submission and **will appear in the published article** if the submission is accepted. Please make sure it is accurate and that any funding sources listed in your Funding Information later in the submission form are also	The authors have declared that no competing interests exist.

declared in your Financial Disclosure statement.<br><br>View published research articles from *PLOS ONE* for specific examples.<br><br>**NO authors have competing interests**<br><br>Enter: *The authors have declared that no competing interests exist.*<br><br>**Authors with competing interests**<br><br>Enter competing interest details beginning with this statement:<br><br>*I have read the journal's policy and the authors of this manuscript have the following competing interests: [insert competing interests here]*<br><br>* typeset	
**Ethics Statement**<br><br>Enter an ethics statement for this submission. This statement is required if the study involved:<br><br>• Human participants<br>• Human specimens or tissue<br>• Vertebrate animals or cephalopods<br>• Vertebrate embryos or tissues<br>• Field research<br><br>Write "N/A" if the submission does not require an ethics statement.<br><br>General guidance is provided below. Consult the submission guidelines for detailed instructions. **Make sure that all information entered here is included in the Methods section of the manuscript.**	N/A

Powered by Editorial Manager® and ProduXion Manager® from Aries Systems Corporation

**Format for specific study types**<br><br>**Human Subject Research (involving human participants and/or tissue)**<br>• Give the name of the institutional review board or ethics committee that approved the study<br>• Include the approval number and/or a statement indicating approval of this research<br>• Indicate the form of consent obtained (written/oral) or the reason that consent was not obtained (e.g. the data were analyzed anonymously)<br><br>**Animal Research (involving vertebrate animals, embryos or tissues)**<br>• Provide the name of the Institutional Animal Care and Use Committee (IACUC) or other relevant ethics board that reviewed the study protocol, and indicate whether they approved this research or granted a formal waiver of ethical approval<br>• Include an approval number if one was obtained<br>• If the study involved *non-human primates*, add *additional details* about animal welfare and steps taken to ameliorate suffering<br>• If anesthesia, euthanasia, or any kind of animal sacrifice is part of the study, include briefly which substances and/or methods were applied<br><br>**Field Research**<br><br>Include the following details if this study involves the collection of plant, animal, or other materials from a natural setting:<br>• Field permit number<br>• Name of the institution or relevant body that granted permission	
**Data Availability**<br><br>Authors are required to make all data underlying the findings described fully available, without restriction, and from the time of publication. PLOS allows rare exceptions to address legal and ethical concerns. See the PLOS Data Policy and FAQ for detailed information.	Yes - all data are fully available without restriction

Powered by Editorial Manager® and ProduXion Manager® from Aries Systems Corporation

A Data Availability Statement describing where the data can be found is required at submission. Your answers to this question constitute the Data Availability Statement and **will be published in the article**, if accepted.<br><br>**Important:** Stating 'data available on request from the author' is not sufficient. If your data are only available upon request, select 'No' for the first question and explain your exceptional situation in the text box.<br><br>Do the authors confirm that all data underlying the findings described in their manuscript are fully available without restriction?	
**Describe where the data may be found in full sentences. If you are copying our sample text, replace any instances of XXX with the appropriate details.**<br><br>• If the data are **held or will be held in a public repository**, include URLs, accession numbers or DOIs. If this information will only be available after acceptance, indicate this by ticking the box below. For example: *All XXX files are available from the XXX database (accession number(s) XXX, XXX.*).<br>• If the data are all contained **within the manuscript and/or Supporting Information files**, enter the following: *All relevant data are within the manuscript and its Supporting Information files.*<br>• If neither of these applies but you are able to provide **details of access elsewhere**, with or without limitations, please do so. For example:<br><br>*Data cannot be shared publicly because of [XXX]. Data are available from the XXX Institutional Data Access / Ethics Committee (contact via XXX) for researchers who meet the criteria for access to confidential data.*<br><br>*The data underlying the results presented in the study are available from (include the name of the third party*	All sequence data has been uploaded to NCBI, with accession numbers provided. The sequencing data will be made public upon manuscript publication. Accession numbers PX642706 - PX642796, PX648620 - PX649011

and contact information or URL).<br>• This text is appropriate if the data are owned by a third party and authors do not have permission to share the data.<br><br>* typeset	
Additional data availability information:	Tick here if the URLs/accession numbers/DOIs will be available only after acceptance of the manuscript for publication so that we can ensure their inclusion before publication.

Powered by Editorial Manager® and ProduXion Manager® from Aries Systems Corporation

Manuscript
Click here to access/download;Manuscript;Cotesia phylogeny manuscipt_14_1_2026.docx

Progress in the molecular phylogeny of *Cotesia acuminata* and *C. melitaearum* cryptic species complexes

Julian Schach[1], Federica Valerio[1], Anne Duplouy[1,2]

[1] Department of Organismal and Evolutionary Biology, Faculty of Biological and Environmental Sciences, the University of Helsinki, Helsinki, Finland

2 Institute of Life Sciences (HiLIFE), the University of Helsinki, Helsinki, Finland

Julian.schach@helsinki.fi, Federica.valerio@helsinki.fi, anne.duplouy@helsinki.fi

Corresponding Author – Julian Schach, Julian.schach@helsinki.fi, Viikinkaari 1, 00790 Helsinki

Author Contributions

JS – Investigation, data curation, methodology, analysis, writing – original draft, writing – review and editing. FV – Methodology, supervision, writing – review and editing. AD – Conceptualization, funding acquisition, project administration, supervision, writing – review and editing.

Keywords: Parasitoids, Maximum likelihood, Melitaeini, Systematics, Braconidae

Abstract

Cryptic species present major challenges for biodiversity and evolutionary research due to the morphological similarity and frequent lack of available genetic and ecological data. In parasitoids wasps, this is especially true for lineages with limited sampling and unclear species boundaries. Here we reconstruct the phylogeny of *Cotesia* wasps parasitizing Melitaeini butterflies, including two cryptic species complexes (*Cotesia acuminata agg.* and *C. melitaearum agg.*). Using a ten-gene dataset from samples collected in Europe, Asia, and North America, we inferred relationships among 22 *Cotesia* species using maximum likelihood. We also included non-Melitaeini associated *Cotesia* to assess whether the parasitoids of Melitaeini form a monophyletic group. Our analyses yielded a highly supported phylogeny, revealing four major clades, three of which included the Melitaeini associated species. This confirms that the *Cotesia* parasitizing Melitaeini butterflies are polyphyletic, resulting from independent host shifts across the genus. Each clade is further subdivided into subclades corresponding to the different cryptic species complexes, clarifying previously unresolved relationships. These results provide a robust framework for future studies on the evolution, ecology, and host use dynamics of *Cotesia* wasps and highlight the utility of multi-locus data for resolving phylogenies in morphologically cryptic taxa.

Introduction

Species have historically been described using morphological data, and species relationships based on degrees of similarities of morphological characteristics. More recently, the advent of molecular techniques has refined many taxonomic/phylogenetic classifications, by allowing us to study species relationships and biodiversity with much higher resolution (1). Cryptic species, are two or more molecularly distinct species, often accompanied by rapid speciation, which are or have been erroneously classified as a single species because they are morphologically indistinguishable (2). Such cryptic species are known to span the tree of life (3–6). Their hidden genetic diversity has however challenged our ability to estimate the true biodiversity richness on Earth, as many remain to be discovered and characterized (7). Furthermore, with the current biodiversity crisis, we are likely losing many such species at alarming rates even before they have been taxonomically characterized, or before their ecosystem roles were understood (8).

Parasitoid wasps make up one of the most diverse animal groups in the world (9). They lay their eggs within or on their hosts' body, and the parasitoid larvae develop feeding on the host, until emergence of the adult wasp (10). These parasitoids can be very efficient at killing their hosts

and can act as key evolutionary pressures on their host species population growth (11) and host population dynamics (12). For example, (12) showed that the specialist species *Cotesia melitaearum* (Wilkinson), parasitizes only 10% of *Melitaea cinxia* (Linnaeus) caterpillars but can still cause localized extinctions within the butterfly host metapopulation. Because of their narrow host ranges and highly efficient killing of their hosts, several species of parasitoids have become popular biocontrol agents in sustainable agriculture, contributing significantly to the $417 billion USD global value of insect biocontrol (Brock et al. 2021). For example, *Cotesia ruficrus* (Haliday), parasitizes the fall armyworm (*Spodoptera frugiperda,* Smith), which is contributing to food insecurity in Africa, Asia, and the Pacific (13)

Most of the worlds parasitoid diversity occurs within the superfamily Ichneumonoidea, which includes the two families Ichneumonidae (Latreille), and Braconidae (Nees von Esenbeck), with approximately 60,000 described species, and many more estimated (14). Within the Braconidae, the subfamily Microgastrinae is the most diverse, with the genus *Cotesia* (Cameron) alone including over 300 named species (Fernandez-Triana et al. 2020), and an estimated 1500-2000 species worldwide (16). The *Cotesia* wasps usually exhibit narrow host ranges, and closely related species often differ in their host species preferences, attacking one to few closely related lepidopteran host species (17). For example, although previous studies suggested that seven species of *Cotesia* were generalist parasites of the Melitaeini (Newman) butterfly species found across Europe and Asia (18,19), more recent molecular analyses accompanied by rearing experiments provided evidence that the originally considered generalist parasitoid species, *Cotesia acuminata* (Reinhard), and *C. melitaearum*, were in fact numerous cryptic species, each with narrow Melitaeini butterfly host specificity (17,20).

In the complex species community of Melitaeini butterflies occurring in Catalonia in northeastern Spain (Catalan Butterfly Monitoring Scheme, CMBS), (17) indeed identified that the 10 local Melitaeini butterfly species, are hosts to at least 10 *Cotesia* (cryptic) species. Unfortunately, the

original barriers to geneflow between the current cryptic lineages of the wasps remain unclear, as studies exploring whether isolation by distance or endosymbiotic bacteria failed to explain the sympatric speciation of the parasitoid species (17,21,22). Currently, the phylogeny of the Catalonian *C. acuminata* complex and *C. melitaearum* complex are based on few microsatellites (17), while a wider Eurasian phylogeny of these groups combined two mitochondrial DNA (mtDNA) loci and a nuclear DNA (nDNA) locus (18). As a consequence of the restricted available genetic material included in these phylogenies, the relationships between *Cotesia* species still contain several unresolved nodes (i.e., nodes with low bootstrap support estimates; Kankare and Shaw 2004, Kankare et al. 2005b), and we still lack a clear description of the diversity of and the speciation scenarios between *Cotesia* wasps attacking Melitaeini butterflies.

Here, we aimed to refine the phylogeny of *Cotesia* species associated with 16 species of Melitaeini butterflies, by using a larger number of nuclear markers and a wider sample diversity than previous studies. We analysed sequences of two mtDNA and eight nDNA markers from both freshly collected specimens from Finland, Italy, Spain, and Switzerland, as well as DNA extracts from samples collected between 1979-2002 from China, Finland, France, Hungary, Russia, Spain, Sweden, and USA (18). With this, we expected to resolve the previously unclear nodes in the *Cotesia* phylogeny, particularly the basal nodes, and to provide a strong phylogenetic reconstruction within the cryptic species complexes.

Materials and Methods

Samples

This study includes *Cotesia* parasitoid wasp specimens that had emerged from the caterpillars of 16 species of Melitaeini butterflies. Including *Euphydryas aurinia* (Rottemburg) *Euphydryas aurinia davidi* (Wahlurg)*,* *Euphydryas cynthia* (Dennis and Schiffermueller)*,* *Euphydryas*

desfontainii (Godart), *Euphydryas editha* (Boisduval), *Euphydryas maturna* (Linnaeus), *Euphydryas phaeton* (Drury), *Melitaea athalia* (Rottemburg), *Melitaea cinxia, Melitaea deione* (Geyer), *Melitaea didyma* (Esper), *Melitaea latonigena* (Eversmann), *Melitaea parthenoides* (Keferstein), *Melitaea phoebe* (Dennis and Schiffermueller), *Melitaea scotosia* (Butler), and *Melitaea trivia* (Dennis and Schiffermueller). (Table 1).

Among the *Cotesia* wasps, two cryptic species complexes are known to consist of multiple species: *C. acuminata* and *C. melitaearum*. Following the convention introduced by (20), we refer to these as *C. acuminata agg.* and *C. melitaearum agg.,* with individual lineages denoted by species-level designations (e.g. *Sp. A., Sp. B.*)

The Melitaeini caterpillars were collected over two time periods: 1979-2002 and 2022-2023. The DNA from early samples collected for the study of *Cotesia* wasps' phylogeny and butterfly-host specialization in earlier studies was extracted in the early 2000's and preserved in the -20 °C freezers at the University of Helsinki (17,18,20). These DNA extracts represent 10 species from eight different countries, including China (N=3 from 1 species), Finland (N=5 from 1 species), France (N=7 from 3 species), Hungary (N=3 from 1 species), Russia (N=6 from 2 species), Spain (N=2 from 1 species), Sweden (N=3 from 1 species), and USA (N=14 from 2 species) (Table 1). Additionally, caterpillars from one locality in Finland (Åland) were also collected in the fall of 2022. Finally, parasitized Melitaeini caterpillars were collected in early April 2023 from five localities in Catalonia, Spain (Coll d'Estenalles, El Brull, Fígols-El Forn, Pyrenees), two localities in Switzerland (Vaud and Valais), and one locality in Italy (Aosta). After collection, the caterpillars were reared in the laboratory on excess of their respective host plant, until the adult wasps emerged. Freshly emerged adult wasps were killed by placing them in a freezer for 24 hours. The samples were stored in 90% ethanol at -20℃ until further manipulated.

Table 1. *Cotesia* identity, host species, number of individuals, caterpillar host plant, country, locality, collector ID, and finally collection year. "-" indicates the information was unavailable.

Cotesia species	Butterfly host species	# of Ind.	Identified host plant	Country	Locality	Collector	Collection year
C. acuminata Sp. A	*Melitaea latonigena*	3	-	Russia	Siberia	N.W. I.H.	1998
C. acuminata Sp. A	*Melitaea didyma*	5	*Plantago lanceolata*	Spain	Coll d'Estenalles	F.V.	2023
C. acuminata Sp. A	*Melitaea didyma*	5	-	Switzerland	Conthey	Y.C.	2023
C. acuminata Sp. B	*Melitaea scotosia*	3	-	China	Peking	I.H., L.G.	1999
C. acuminata Sp. B	*Melitaea phoebe*	5	-	Italy	Aosta	Y.C.	2023
C. acuminata Sp. K	*Euphydryas maturna*	1	-	France,	Cote d'Or, Malloy, Lindesberg	P.J.C.R.	1999
C. acuminata Sp. K	*Euphydryas maturna*	3	-	Sweden	Lindesberg	C.U.E.	1999
C. acuminata Sp. L	*Melitaea athalia*	1	-	France	Var, St. Paul-en-Foret	P.W.C.	1982
C. bignellii	*Euphydryas aurinia*	5	-	Switzerland	La Vraconnaz	Y.C.	2023
C. cynthiae	*Euphydryas cynthia*	4	-	France	Alps, Laus de Cervieres	M.S.	2001
C. euphydryidis	*Euphydryas phaeton*	7	-	USA	Warren Co., Front Royal	N.S.	1979
C. koebelei	*Euphydryas editha*	7	-	USA	Yosemite National Park	M.S., B.W.	2001
C. melitaearum Sp. D	*Euphydryas desfontainii*	5	*Cephalaria leucantha*	Spain	Fígols-El Forn	CS	2023

Cotesia species	Butterfly host species	# of Ind.	Identified host plant	Country	Locality	Collector	Collection year
C. melitaearum Sp. D	*Euphydryas aurinia*	5	*Lonicera implexa*	Spain	Cornudella de Montsant	C.S.	2023
C. melitaearum Sp. D	*Euphydryas aurinia*	5	*Lonicera implexa*	Spain	La Mussara	C.S.	2023
C. melitaearum Sp. E	*Melitaea deione*	5	*Antirhinum majus*	Spain	Les Illes	CS	2023
C. melitaearum Sp. E	*Melitaea deione*	5	*Antirhinum majus*	Spain	Poboleda	CS	2023
C. melitaearum Sp. F	*Melitaea didyma*	3	-	Hungary	Örseg	M.R.S.	2001
C. melitaearum Sp. F	*Melitaea didyma*	2	-	Spain	Cantallops	C.S.	2002
C. melitaearum Sp. G	*Melitaea trivia*	10	*Verbascum pulverulentum*	Spain	Pyrenees	C.S.	2023
C. melitaearum Sp. H	*Melitaea cinxia*	8	*Plantago lanceolata*	Finland	Åland	S.I.	2022
C. melitaearum Sp. I	*Melitaea athalia*	5	-	Finland	Åland	L.G., S.vN.	1997
C. melitaearum Sp. M	*Euphydryas aurinia davidi*	3	-	Russia	Siberia	N.W., I.H.	1998
C. melitaearum Sp. N	*Melitaea parthenoides*	5	*Plantago media*	Spain	El Brull	C.S.	2023

Collectors: Y.C., Yannick Chitarro; P.W.C, Peter W. Cribb; C.U.E., Clae U. Eliasson; L.G., Lei Guanchung; I.H., Ilkka Hanski; S.I., Suvi Ikonen: P.J.C.R., Peter J. C. Russel: M.R.S., Mark R. Shaw; M.S., Michael Singer; N.S., Nancy Stamp; C.S., Constanti Stefansescu; S. vN., Saskya van Nouhys; N.W. Niklas Wahlberg; B.W., Brian Wee; F.V. Federica Valerio

To test whether the *Cotesia* species parasitizing Melitaeini butterflies form a monophyletic group, we also included gene sequences retrieved from *Cotesia chilonis* (Munakata)*, Cotesia congregata* (Say)*, Cotesia glomerata* (Linnaeus)*, C. ruficrus, Cotesia typhae* (Fernández-Triana)*,* and *Cotesia vestalis* (Haliday)*,* which full genomes are publicly available from the NCBI database. These *Cotesia* species naturally parasitize species of pest moths (e.g. *Spodoptera*

(Guenée), or *Manduca* (Hübner)) or *Peiris* (Schrank) butterflies (23,24). We also included respective gene sequences from two Microgastrine species (*Microplitis demolitor* (Wilkinson), and *Microplitis mediator* (Haliday)), which full genomes are publicly available from the NCBI database (Table 2), to use as outgroup in the phylogenies described below. Our sequences were blasted against the respective genomes to extract the sequences of the genes used in the phylogeny. All NCBI references can be found in Table 2.

Table 2. Host species, GenBank accession number, and reference for seven species added to the *Cotesia* ingroup and *Microplitis* outgroup of the phylogenetic tree. "-" indicates the information was unavailable.

Cotesia Species	Host	GenBank accession #	Reference
Cotesia chilonis	*Chilo suppresalis*	GCA_018835575.1	(25)
Cotesia congregata	*Manduca sexta*	GCA_905319865.3	(26)
Cotesia glomerata	-	GCA_020080835.1	(27)
Cotesia ruficrus	*Mythimna separata*	GCA_048537615.1	-
Cotesia typhae	*Sesamia nonagrioides*	GCA_013202065.2	(28)
Cotesia vestalis	*Plutella xylostella*	GCA_000956155.1	-
Microplitis demolitor	*Pseudoplusia includens*	GCA_026212275.2	(29)
Microplitis mediator	-	GCA_029852145.1	-

Depending on availability, we selected one to seven (on average five) parasitoid specimens per caterpillar species, and per locality sampled. As expected, the cryptic nature of the parasitoids, and the fact that some caterpillar species can host multiple species of parasitoids makes their identification difficult using only morphology. The species identity of the parasitoids collected in the early 2000's was previously confirmed through morphology, *COI,* and host associations (18). For the samples collected in 2022 and 2023, their species identities were assigned after blasting their respective *COI* sequences against the NCBI database, and by considering their host associations, following the protocol used by (18)

DNA Extraction

For the samples collected in the early 2000’s the DNA was extracted using the NucleoSpin Tissue Kit (Macherey-Nagel) following the manufacturers protocol and eluted in 50 µL of MilliQ water (18). The samples have since been stored in the freezer (-20℃) at the University of Helsinki, Finland. For the samples collected in 2022 and 2023, the DNA was extracted from each whole insects, except in two cases where we pooled adult wasps that emerged from the same host caterpillar. The Macherey-Nagel NucleoSpin Tissue Kit (Düren, Germany) was used following the manufacturer’s protocol. The DNA was eluted twice in 40 µL of elution buffer to increase final yield and concentration.

Gene selection

Genetic markers and primer pairs designed and tested by previous studies, were first selected by reviewing the available literature on *Cotesia* and Braconidae phylogenies (See Table 2. for references). All primer pairs were tested for specificity to *Cotesia* using Primer-Blast (30). Additionally, 16 BUSCO genes were randomly selected from the *C. congregata* genome (GCA_905319865.3) using BUSCO v 5.7.1 (31). Using Primer-Blast (30), we designed an additional set of 16 primer pairs to amplify these 16 BUSCO genes. The best pairs were selected based on the lowest self-complimentary score given by Primer-Blast. In total two mtDNA markers and 23 nDNA markers were tested, but only two and eight, respectively, were successfully used in this study, as 13 other nDNA primer pairs designed failed to amplify through gradient PCR (Table S5). All primer pairs, from the literature and designed for this study, targeted genetic regions with a length ranging from 150 to 850 bp. All primer pairs used in the present study are listed in Table 3.

Table 3. Characteristics of the primer pairs used in this study. Including the name of the marker targeted (gene), primer name, primer sequence, amplicon size, annealing temperature, and source. “-” indicates the information was unavailable.

Gene	Primer name	Sequence (5'-3')	Amplicon size	Ta (℃)	Primer source
COI	LCO2198	5`- GGTCAACAAATCATAAAGATATTGG	658	49	Folmer et al., 1994
	HCO1490	5`- TAAACTTCAGGGTGACCAAAAAATCA			
16s	16SWb	5`- CACCTGTTTATCAAAAACAT	375	58	Dowton and Austin, 1994; Whitfield, 1997
	16S outer	5`- CTTATTCAACATCGAGGTC			
18s	18s - F	5`- CATTGGAGGGCAAGTCTGGTGCCA	240	58	Quicke et al. 2020
	18s - R	5`- AGTAAACGTACCGGCCCTCCTCG			
n28s rDNA	28s	5`- AAGAGAGAGTTCAAGAGTACGTG	618	56	Mardulyn & Whitfield, 1999
	28s-PM	5`- TAGTTCACCATCTTTCGGGTCCC			
EF1a	EF1A1 - F	5`- AGATGGGYAARGGTTCCTTCAA	418	56	Belshaw and Quike, 1997
	EF1A1 - R	5`- AACATGTTGTCDCCGTGCCATCC			
ITS2	NG02955	5`- ATGAACATCGACATTTCGAACGCACA	-	60	Regueira et al. 2017
	AB052895	5`- TTCTTTTCCTCCGCTTAGTAATATGCTTAA			
LW-Rh	OpsFor2	5`-GGATGTASCTCCATTTGGTC	-	58	Banks and Whitfield, 2006
	Ops3'2	5`-AVHGATGCRACRTTCATTTTCT			
Elob	TCEB - F	5`-TCTTAAAAATTCCTCCAACGCATC	250	57	This study
	TCEB - R	5`-GATGCCTGCTCTTGTCCATT			
SLD5	SLD5 - F	5`-CCTAAGAACGCGGTTGGAGA	327	57	This study
	SLD5 - R	5`-TGAACAATATGCTGCGAGCC			

Innexin	INX-F	5`-CGCTCTTTGTGTGCTTGCTT	342	54	This study
	INX-R	5`-GGTACAGAAGTCGGTGAGGC			

PCR

Each 10 µL PCR reaction included 1 µL of Buffer solution, 0.8 µL of DNTPs (25nM) mix, 0.5 µL of forward primers (10nM), 0.5 µL of reverse primers (10nM), 0.5 µL Mg2 reagent, 0.05 µL KAPA Taq polymerase, 6.7 µL of H20 (sterile), and 1 µL of DNA extract. The PCR reactions were initiated by incubation at 95°C for 3 min, followed by 30 cycles of 95°C for 40 s, the optimal annealing temperature of the respective primer pair (Table 2) for 40 s, and 72°C for 40 s. The PCR reaction ended with a final elongation step at 72°C for 5 min, and a final hold at 18°C.

The PCR products were run through a 1% agarose gel electrophoresis, with SYBR-safe stain (Thermo Scientific, USA), and visualized under UV light. For each gel, 5 µL of 100 bp DNA ladder (#SM0321, Thermo Scientific, USA) was loaded into the first well, and each subsequent well was loaded with a mixture of 4 µL of both loading dye (bromophenol blue) and PCR product. The gels were run for 30 minutes at 100 volts. The PCR amplification was repeated once for any failed sample, to identify potential false negative results.

Preparation for Sanger Sequencing

The successfully amplified PCR products were individually cleaned using Exonuclease I (ExoI) to remove any single-stranded DNA (Thermo Scientific, USA). For each reaction 0.5 µL of ExoI was added to 5 µL of PCR product, then the samples were incubated at 37°C for 15 minutes, followed by 15 minutes at 85°C to terminate the reaction.

Cleaned PCR products were sent to Macrogen Europe for Sanger Sequencing using the company Multiple Primer/Plate option.

Sequence Cleaning and Alignments

The chromatograms of each sequence were manually quality checked by using Geneious Prime 2025.01 (https://www.geneious.com). Low-quality sequences (error probability limit 0.05) and highly incomplete sequences (less than half of target length) were removed from further analysis.

We included the respective gene sequences from two Microgastrine species (*Microplitis demolitor,* and *Microplitis mediator*), to use as outgroup in the phylogenies described below. Additionally, six species of *Cotesia* that do not parasitize Melitaeini were added to the ingroup (Table 2). For each maker, all sequences were aligned with MUSCLE (32), and all alignments were manually curated with AliView (33). The accession numbers for the sequences submitted to NCBI are PX523132-PX523219, PX559195-PX559573, PX642706-PX642796, and PX648620-PX649011

Cotesia Phylogeny

For the phylogenetic analysis we used maximum likelihood (ML) with IQ-TREE V2.4.0 (34). Phylogenetic analyses were conducted for: (I) all ten genes concatenated (mtDNA and nDNA), (II) eight concatenated nuclear genes, (III) two concatenated mitochondrial genes, and (IV) all genes independently. Specimens that amplified for less than half of the markers were excluded from concatenated analysis but kept for the individual gene tree analyses. Phylogenetic trees were visualized using ITOL (35).

For ML analysis, we used ModelFinder (36), and the Bayesian Information Criterion (BIC) to select for the best models. The partition finding algorithm (37) was run for the concatenated data (ten-genes, nDNA, mtDNA) partitioned by genes using the MFP+MERGE option. Support for nodes was evaluated with 1000 ultrafast bootstrap (UFBoot) approximations (38).

Results

Parasitoid Identification

The N=42 specimens collected from 1979 to 2002 include 10 species of *Cotesia*: four cryptic species of *C. acuminata agg*, *Cotesia cynthiae* Nixon 1974*, Cotesia euphydryidis, Cotesia koebelei* Riley, 1889*,* and three cryptic species of *C. melitaearum*, either as previously identified by earlier studies (17,18) or by comparing their COI sequences to NCBI. The sequencing results of the 2022 and 2023 collections confirmed that these N=68 specimens include eight species of *Cotesia*: *Cotesia bignellii* Marshall, 1885, two cryptic species of *C. acuminata agg.*, and five cryptic species of *C. melitaearum agg.*

Sequencing results

Of 110 specimens sequenced for this study, 41% were successfully sequenced for all 10 genes, 66% for at least 9 genes, 75% for at least 8 genes, 85% for at least 7 genes, 88% for at least 6 genes, and 90% for at least 5 genes.

Cotesia Phylogenies

(I) Ten-gene dataset

The ten-gene dataset included 110 wasp specimens (102 Melitaeini associated, and eight from NCBI). The ten-gene alignment length was 3486 bp long, of which 1498 sites (43%) represented unique site-patterns. Out of these, 747 sites (21% of all sites) were parsimony-informative. The model partitioning algorithm revealed five partitions in the ten-gene dataset (Table S1.)

The ML analysis revealed four major clades with high support (bootstrap = 100, 77, 93) and suggested that the species attacking Melitaeini butterflies are polyphyletic (Fig. 1.).

- **Clade A** contains the *C. acuminata agg., C. bignellii,* and *C. euphydryidis* parasitoids from Melitaeini butterflies*. Cotesia euphydryidis* is placed at the base of the clade. The next division is between *C. bignellii* and *C. acuminata agg.* The clade reveals that *C. acuminata agg.* is monophyletic, where *Sp. B* and *K* cluster together and are sister taxa to *Sp. A.* The divergence between the *Sp. A*, and *B/K* is poorly supported (bootstrap = 49), but the divergence between *Sp. B* and *K* is well supported (Fig. 1.).
- **Clade B** contains *C. koebelei, C. congregata* and *C. glomerata* parasitoid wasps. *Cotesia congregata* and *C. glomerata* do not use Melitaeini butterflies as hosts but *C. koebelei* wasps, parasitize the Melitaeini species *E. editha.* The nodes in this clade are highly supported (bootstrap = 98 and above) (Fig. 1.).
- **Clade C** contains *C. chilonis, C. ruficrus, C. typhae,* and *C. vestalis* parasitoids, which all parasitize non-Melitaeini hosts. The divergence between these species is highly supported (bootstrap = 93 and above) (Fig. 1.).
- **Clade D** contains *C. cynthiae* and *C. melitaearum agg,* which parasitize Melitaeini butterflies*.* Ten subclades are revealed, represented by the individual cryptic species and *C. cynthiae* with moderate to high support (bootstrap 77 to 100). Here, *C. melitaearum agg.* forms a paraphyletic group. Most of the individual species within this clade group together, forming monophyletic groups, except *C. melitaearum Sp. F*. For this particular species, our phylogeny places the specimens from Spain in a sister group to *C. melitaearum Sp. G* also from Spain and the specimens from Hungary are in a sister-group to the *C. melitaearum Sp. H* from Finland. (Fig. 1.).

Figure 1. Maximum likelihood phylogeny of *Cotesia* species parasitizing different Melitaeini host species and their relatives based on the ten concatenated mitochondrial and nuclear genes (16s, *18s, 28s, COI, EF1a, INX, ITS2, LW-Rh, SLD5,* and *Elob*)*.* The specimens are labelled by the names of the *Cotesia* species and Melitaeini host caterpillar species. Bootstrap support values (1000 replicates) are indicated for supported branches (≥60). Colors represent the main clades (A, B, C, D). Branches corresponding to the same species were collapsed for visual clarity and represented by triangles.

(II) Nuclear-gene dataset

The nuclear-gene dataset included 112 wasp specimens (104 Melitaeini associated, and eight from NCBI). The concatenated sequence was 2413 bp long, of which 1035 sites (43%) represented unique site-patterns, and of these 494 (20% of all sites) were parsimony informative. The model partitioning algorithm revealed five partitions in the nuclear-gene dataset (Table S2).

The ML analysis produced a highly supported phylogeny that is largely congruent with the ten-gene tree. The same four major clades were recovered with strong support (bootstrap = 100, 82, 87) with key differences being the topology within clades A and D (Fig. 2.).

- **Clade A** now groups *C. acuminata Sp. B* emerging from *M. scotosia* with *C. acuminata Sp. K.* Again, the relationships between the cryptic species have lower support (bootstrap = 60) (Fig. 2.).
- **Clade D** shows some discordance from the ten-gene phylogeny. For example, *C. melitaearum Sp. F* is grouped together with *C. melitaearum Sp. H* and is no longer polyphyletic, thus resulting in nine subclades. Additionally, *C. cynthiae* and *C. melitaearum Sp. M* are no longer sister groups. Meanwhile, the relationships between *C. melitaearum Sp. N, E* and *D* are concordant with the ten-gene dataset.

Figure 2. Maximum likelihood phylogeny of *Cotesia* species parasitizing different Melitaeini host species and their relatives based on the nuclear genes (*18s, 28s, EF1a, INX, ITS2, LW-Rh, SLD5,* and *Elob*). The specimens are labelled by the names of the *Cotesia* species and Melitaeini host caterpillar species. Branches corresponding to the same species were collapsed for visual clarity and represented by triangles. Bootstrap support values (1000 replicates) are indicated for supported branches (≥60). Colors represent the main clades (A, B, C, D).

(III) Mitochondrial-gene dataset

The mitochondrial-gene dataset included the sequences of the *16s* and *COI* genes from 92 specimens (86 Melitaeini associated, six from NCBI). The mitochondrial alignment was 1072bp long, of which 451 (42%) represented unique site-patterns, of which 242 sites (23% of all sites) were parsimony informative. The model partitioning algorithm revealed two partitions in the mitochondrial gene dataset, which correspond to the two markers (Table S3). No specimens from *C. euphydryidis* and *C. melitaearum Sp. F* from Hungary amplified for either mitochondrial marker, and no sequences were found from *C. typhae* and *C. vestalis* genomic projects, thus they are missing from the mitochondrial phylogenetic analysis.

The ML analysis revealed the same four major clades as the ten-gene and nuclear gene phylogenies, however with varying support (bootstrap = 100, 49, 97) and topology.

- **Clade B** now forms the basal clade of the phylogeny. The relationships within the clade exposed in the ten-gene and nuclear phylogenies are conserved.
- **Clade A** with **Clade C** and **Clade D** now group together as sister taxa to Clade B**.** Clades C and D are sister clades to Clade A, which is congruent the ten-gene phylogeny. The relationships within Clade A and C are congruent to the ten-gene phylogeny, despite missing species. The ~~relationships~~ between *C. acuminata agg.* species is low (bootstrap = 60). Within clade D, we can still observe the paraphyletic nature of this cryptic complex, forming nine subclades comprised of the *C. melitaearum agg.* and *C. cynthiae*. Additionally, *C. melitaearum Sp. D* does not cluster by the two hosts. Many of the relationships in this clade are unsupported (bootstrap < 60) (Fig. 3.).

Figure 3. Maximum likelihood phylogeny of *Cotesia* species parasitizing different Melitaeini host species and their relatives based on the mitochondrial genes (*16s* and *COI*). The specimens are labelled by the names of the *Cotesia* species and Melitaeini host caterpillar species. Branches corresponding to the same species were collapsed for visual clarity and represented by triangles. Bootstrap support values (1000 replicates) are indicated for supported branches (≥60). Colors represent the main clades (A, B, C, D).

(IV) Individual Genes

All genes were also analysed individually using ML to identify discordance or outlier genes. The substitution models for the ML analyses are listed in Table S4. Rather expectedly, the individual gene phylogenies had much lower branch supports than either of the other phylogenies, likely because of the small nucleotide sample size included in each analysis. Alone, the individual gene trees are not phylogenetically informative. The major clades were only recovered in the *16s*, *COI*, and *SLD5* ML trees, but the relationships within the clades, particularly within the Clade C, remain incongruent (Fig. S1-S10.).

Discussion

Resolving species phylogenies is essential for studying trait evolution, diversification, and ecological interactions, but can remain challenging in non-model cryptic species, due to limited access to sampling and molecular resources. By designing new primers and increasing the number of available molecular markers from two to ten, we were able to produce the most robust phylogeny of the *Cotesia* species parasitizing Melitaeini butterflies to date. This improved phylogeny informs on previously hidden species diversity within this genus and is an important resource to future research on Braconidae population genetics, evolutionary dynamics and ecology.

Our analyses revealed that all specimen’s group in four highly supported clades (A-D), each subdivided into distinct subclades of (cryptic) species. By including genetic sequences from *Cotesia* species that do not attack Melitaeini butterflies, we confirm that the Melitaeini-specialized species are polyphyletic, appearing in three of the four main *Cotesia* clades. This suggests that *Cotesia* wasps have shifted hosts multiple times throughout their evolutionary history. Such patterns of host shifts have been observed in other parasitoid lineages, including Ichneumonoidea (39), and parasitoids of leaf miners (40), indicating that parasitoid evolution often involves ecological switching rather than strict co-divergence with hosts.

Many of these Melitaeini-associated *Cotesia* species occur in sympatry, yet the drivers of radiation and barriers to gene flow remain unclear (17,21). Host evolution appears to play a partial role. For example, in Clade A, the basal species parasitize *Euphydryas* caterpillars, while most *C. acuminata* species have shifted to *Melitaea.* Given that *Euphydryas* butterflies diverged before *Melitaea* (41), it likely supports the hypothesis that the ancestral host for Clade A belonged to the *Euphydryas* butterfly lineage. A similar pattern may exist in Clade B where *C. koebelei* parasitizes *E. editha* and has also been tentatively reported from *Melitaea* (42). If *C. koebelei* parasitizes both *Euphydryas* and *Melitaeini* butterflies, it could represent a cryptic species complex, or a rare generalist able to attack both butterfly genera. The question on which of these two hypotheses is the most plausible will only be resolved through future intensive sampling of North American Melitaeini butterflies and their *Cotesia* parasitoids.

In contrast, Clade D shows an opposite pattern to Clade A: with basal species parasitizing *Melitaea*, followed by a shift to *Euphydryas*. This may reflect a retained ability to use *Euphydryas* inherited from a shared ancestor with Clade A, possibly representing a recolonization event. Species from Clade C are not known to use Melitaeini butterflies but span a wider range of Lepidoptera host taxa. For example, *C. vestalis* parasitizes *Plutella xylostella,* (Linnaeus, 1758) (Yponomeutoidea), a lineage near the base of the Lepidopteran tree (43), while more recently diverged *Cotesia* in this clade parasitize more recently diverged groups such as Pyraloidea, (Latreille, 1802) and Noctoidae, (Latreille, 1809).

Although the relationships within and between cryptic species were better supported in our final phylogeny compared to previous studies (18), comparisons of the tree topologies suggested conflicts between nuclear and mitochondrial reconstructions. The discordance within clades may reflect biological processes such as geneflow between lineages or rapid radiation resulting in incomplete lineage sorting (44,45). The differences between methods and datasets might be

due to the non-random absence of mitochondrial data for several taxa in two clades, which can bias phylogenetic inference, especially when relatively few markers are used (46).

While we bring an improved version of the *Cotesia* phylogeny, our data still remains too weak to fully evaluate the level of hybridization and introgression between the lineages, or the frequency of host shifts within clades. Additionally, our restricted dataset could have mislead phylogenetic inference especially under incomplete lineage sorting (46). In the future, the use of additional genetic material, specimens from other species, or a wider geographic range will be needed to confidently evaluate the extent of the true evolutionary history of these events.

Such increased sampling would also allow for explicit research on the contributions of geography, ecology, and host associations in the diversification of *Cotesia*. For example, other parasitoid lineages show patterns of latitudinal diversity gradients and geographic structuring (47) and similarities might be revealed in *Cotesia*. Additionally, the butterfly host ecology could be explored, which has been observed in parasitoids of leaf miners that often radiate to unrelated hosts that share the same host plant (Leppänen et al., 2012). Finally, studying host associations and range may also influence *Cotesia* diversity through mechanisms such as host fidelity. Whereby many parasitoid wasp females preferentially oviposit on the same host species from which they emerged, reinforcing reproductive barriers and potentially promoting speciation (Forbes et al., 2017; Hood et al., 2015; Kimura and Novković, 2015). Network based approaches provide promising tools to study host-parasitoid coevolution (Braga et al., 2018), testing hypotheses such as adaptive radiation (Ehrlich and Raven, 1964), or host use oscillations (Janz and Nylin, 2008). Understanding host repertoire has important implications for conservation, as species with narrow fundamental host ranges face greater extinction risks, and identifying such species can help prioritize conservation efforts (Farrell et al., 2021).

Conclusion

Out study provides a robust phylogenetic framework for *Cotesia* parasitizing Melitaeini butterflies, clarifying uncertainties in their evolutionary relationships. The results show that the species attacking Melitaeini are paraphyletic, grouping in three of the four major clades, indicating independent host shifts. Although our sampling does not allow for inference of which lineage first adapted to Melitaeini hosts, the confirmation of distinct cryptic species in *C. acuminata agg* and *C. melitaearum agg.* highlights the extent of hidden diversity in this system. Together these findings establish a foundation for future taxonomic revisions, comparative genomic studies, and exploration of the ecological barriers that initiated and maintain their reproductive isolation.

Data Availability

NCBI accession numbers for all markers and specimens will be available upon publication (accession numbers have been created for all sequences, cited in the manuscript). The authors are happy to share the sequence files with reviewers upon request.

Acknowledgements

Thanks to the members of the ISEE lab and life-history evolution group at the University of Helsinki for the many discussions and input. Thank you to Dr. Victoria Twort for her input on the manuscript. Thanks to everyone involved in the sample collection, including Constantí Stefanescu and his team in Spain, Yannick Chittaro in Switzerland, and in Finland Suvi Ikonen and Krista Raveala, along with those involved in the Åland butterfly survey.

References

1. Kjer KM, Simon C, Yavorskaya M, Beutel RG. Progress, pitfalls and parallel universes: a history of insect phylogenetics. J R Soc Interface. 2016 Aug;13(121):20160363.
2. Bickford D, Lohman DJ, Sodhi NS, Ng PKL, Meier R, Winker K, et al. Cryptic species as a window on diversity and conservation. Trends Ecol Evol. 2007 Mar;22(3):148–55.
3. Kon T, Yoshino T, Mukai T, Nishida M. DNA sequences identify numerous cryptic species of the vertebrate: A lesson from the gobioid fish *Schindleria* . Mol Phylogenet Evol. 2007 Jul;44(1):53–62.
4. Shin CP, Allmon WD. How we study cryptic species and their biological implications: A case study from marine shelled gastropods. Ecol Evol. 2023 Sep 5;13(9).
5. Grundt HH, Kjølner S, Borgen L, Rieseberg LH, Brochmann C. High biological species diversity in the arctic flora. Proceedings of the National Academy of Sciences. 2006 Jan 24;103(4):972–5.
6. Hebert PDN, Penton EH, Burns JM, Janzen DH, Hallwachs W. Ten species in one: DNA barcoding reveals cryptic species in the neotropical skipper butterfly *Astraptes fulgerator*. Proceedings of the National Academy of Sciences. 2004 Oct 12;101(41):14812–7.
7. Li X, Wiens JJ. Estimating global biodiversity: The role of cryptic insect species. Syst Biol. 2023 Jun 16;72(2):391–403.
8. Koh LP, Dunn RR, Sodhi NS, Colwell RK, Proctor HC, Smith VS. Species coextinctions and the biodiversity crisis. Science (1979). 2004 Sep 10;305(5690):1632–4.
9. Forbes AA, Bagley RK, Beer MA, Hippee AC, Widmayer HA. Quantifying the unquantifiable: Why Hymenoptera, not Coleoptera, is the most speciose animal order. Vol. 18, BMC Ecology. BioMed Central Ltd.; 2018.
10. Whitfield JB, Austin AD, Fernandez-Triana JL. Systematics, biology, and evolution of Microgastrine parasitoid wasps. Annu Rev Entomol. 2018 Jan 7;63(1):389–406.
11. Cornell H V., Hawkins BA. Patterns of parasitoid accumulation on introduced herbivores. In: Hawkins BA, Sheehan W, editors. Parasitoid Community Ecology. Online. Oxford University Press; 1994. p. 77–89.
12. Lei GC, Hanski I. Metapopulation structure of *Cotesia melitaearum*, a specialist parasitoid of the butterfly *Melitaea cinxia*. Oikos. 1997 Feb;78(1):91.
13. Li X, Jia JJ, An JL, Meng FX, Liu TX, Zhang SZ. Effect of *Cotesia ruficrus* parasitization on diversity and community composition of intestinal bacteria in *Spodoptera frugiperda*. Insects. 2024 Jul 27;15(8):570.
14. Quicke DLJ. The Braconid and Ichneumonid Parasitoid Wasps. Wiley; 2014. 341–449 p.
15. Fernandez-Triana J, Shaw MR, Boudreault C, Beaudin M, Broad GR. Annotated and illustrated world checklist of Microgastrinae parasitoid wasps (Hymenoptera, Braconidae). Zookeys. 2020 Mar 23;920:1–1089.

16. Mason WRM. The polyphyletic nature of *Apanteles Foerster* (Hymenoptera: Braconidae): a phylogeny and reclassification of Microgastrinae. Memoirs of the Entomological Society of Canada. 1981 May 31;113(S115):1–147.

17. Kankare M, Stefanescu C, Van Nouhuys S, Shaw MR. Host specialization by *Cotesia* wasps (Hymenoptera: Braconidae) parasitizing species-rich Melitaeini (Lepidoptera: Nymphalidae) communities in north-eastern Spain. Biological Journal of the Linnean Society. 2005 Sep;86(1):45–65.

18. Kankare M, Shaw MR. Molecular phylogeny of *Cotesia* Cameron, 1891 (Insecta: Hymenoptera: Braconidae: Microgastrinae) parasitoids associated with Melitaeini butterflies (Insecta: Lepidoptera: Nymphalidae: Melitaeini). Mol Phylogenet Evol. 2004 Jul;32(1):207–20.

19. Wahlberg , Niklas, Kullberg J, Hanski I. Natural history of some Siberian melitaeine butterfly species (Nymphalidae: Melitaeini) and their parasitoids. Entomol Fenn. 2001 Jun 1;12(2).

20. Kankare M, Van Nouhuys S, Hanski I. Genetic divergence among host-specific cryptic species in *Cotesia melitaearum Aggregate* (Hymenoptera: Braconidae), parasitoids of checkerspot butterflies. Ann Entomol Soc Am. 2005 May;98(3):382–94.

21. Valerio F, Martel C, Stefanescu C, Duplouy A. *Wolbachia* strain diversity in a complex group of sympatric cryptic parasitoid species. 2023; Available from: https://doi.org/10.21203/rs.3.rs-3649450/v1

22. Schach J. Molecular Phylogeny of *Cotesia* Wasps Parasitizing Melitaeini Butterflies and the Possible Role of *Wolbachia* Endosymbionts [Master's thesis]. [Helsinki]: University of Helsinki; 2024.

23. Miles CI, Chen WP, Adamo SA, Kester KM, Miller DW. *Manduca sexta* caterpillars parasitized by the wasp *Cotesia congregata* stop chewing despite an intact motor system. Journal of Experimental Biology. 2023 Aug 15;226(16).

24. Blažytė-Čereškienė L, Aleknavičius D, Apšegaitė V, Būda V. Response of parasitic wasp *Cotesia glomerata* l. (hymenoptera: braconidae) to cabbage plants of two varieties: olfactory spectra of males and females. J Econ Entomol. 2022 Oct 12;115(5):1464–71.

25. Ye X, Xiong S, Teng Z, Yang Y, Wang J, Yu K, et al. Retraction: Amino acid synthesis loss in parasitoid wasps and other hymenopterans. Elife. 2020 Dec 1;9.

26. Gauthier J, Boulain H, van Vugt JJFA, Baudry L, Persyn E, Aury JM, et al. Chromosomal scale assembly of parasitic wasp genome reveals symbiotic virus colonization. Commun Biol. 2021 Jan 22;4(1):104.

27. Pinto BJ, Weis JJ, Gamble T, Ode PJ, Paul R, Zaspel JM. A chromosome-level genome assembly of the parasitoid wasp, *Cotesia glomerata* (Hymenoptera: Braconidae). Journal of Heredity. 2021 Nov 1;112(6):558–64.

28. Muller H, Chebbi MA, Bouzar C, Périquet G, Fortuna T, Calatayud PA, et al. Genome-wide patterns of bracovirus chromosomal integration into multiple host tissues during parasitism. J Virol. 2021 Oct 27;95(22).

29. Burke GR, Strand MR. Deep sequencing identifies viral and wasp genes with potential roles in replication of *Microplitis demolitor* bracovirus. J Virol. 2012 Mar 15;86(6):3293–306.

30. Ye J, Coulouris G, Zaretskaya I, Cutcutache I, Rozen S, Madden TL. Primer-BLAST: a tool to design target-specific primers for polymerase chain reaction. BMC Bioinformatics. 2012 Jun 18;13:134.

31. Manni M, Berkeley MR, Seppey M, Zdobnov EM. BUSCO: Assessing genomic data quality and beyond. Curr Protoc. 2021 Dec 22;1(12).

32. Edgar RC. MUSCLE: multiple sequence alignment with high accuracy and high throughput. Nucleic Acids Res. 2004 Mar 8;32(5):1792–7.

33. Larsson A. AliView: a fast and lightweight alignment viewer and editor for large datasets. Bioinformatics. 2014 Nov 15;30(22):3276–8.

34. Minh BQ, Schmidt HA, Chernomor O, Schrempf D, Woodhams MD, von Haeseler A, et al. IQ-TREE 2: New models and efficient methods for phylogenetic inference in the genomic era. Mol Biol Evol. 2020 May 1;37(5):1530–4.

35. Letunic I, Bork P. Interactive Tree of Life (iTOL) v6: recent updates to the phylogenetic tree display and annotation tool. Nucleic Acids Res. 2024 Jul 5;52(W1):W78–82.

36. Kalyaanamoorthy S, Minh BQ, Wong TKF, von Haeseler A, Jermiin LS. ModelFinder: fast model selection for accurate phylogenetic estimates. Nat Methods. 2017 Jun 8;14(6):587–9.

37. Chernomor O, von Haeseler A, Minh BQ. Terrace aware data structure for phylogenomic inference from supermatrices. Syst Biol. 2016 Nov;65(6):997–1008.

38. Hoang DT, Chernomor O, von Haeseler A, Minh BQ, Vinh LS. UFBoot2: Improving the ultrafast bootstrap approximation. Mol Biol Evol. 2018 Feb 1;35(2):518–22.

39. Quicke DLJ. We know too little about parasitoid wasp distributions to draw any conclusions about latitudinal trends in species richness, body size and biology. PLoS One. 2012 Feb 15;7(2).

40. Leppänen SA, Altenhofer E, Liston AD, Nyman T. Ecological versus phylogenetic determinants of trophic associations in a plant–leafminer–parasitoid food web. Evolution (N Y). 2012 Dec;no-no.

41. Long EC, Thomson RC, Shapiro AM. A time-calibrated phylogeny of the butterfly tribe Melitaeini. Mol Phylogenet Evol. 2014 Oct;79:69–81.

42. Moore SD. Regulation of host diapause by an insect parasitoid. Ecol Entomol. 1989 Feb 14;14(1):93–8.

43. Kawahara AY, Plotkin D, Espeland M, Meusemann K, Toussaint EFA, Donath A, et al. Phylogenomics reveals the evolutionary timing and pattern of butterflies and moths. Proceedings of the National Academy of Sciences. 2019 Nov 5;116(45):22657–63.

44. Zadra N, Rizzoli A, Rota-Stabelli O. Chronological incongruences between mitochondrial and nuclear phylogenies of *Aedes* mosquitoes. Life. 2021 Feb 25;11(3):181.

45. Maddison WP. Gene Trees in Species Trees. Syst Biol. 1997 Sep 1;46(3):523–36.

46. Xi Z, Liu L, Davis CC. The impact of missing data on species tree estimation. Mol Biol Evol. 2016 Mar;33(3):838–60.

47. Castellanos-Labarcena J, Steinke D, Adamowicz SJ. Anomalous latitudinal gradients in parasitoid wasp diversity—Hotspots in regions with larger temperature range. Journal of Animal Ecology. 2025 Mar 13;94(3):410–22.

48. Kimura MT, Novković B. Local adaptation and ecological fitting in host use of the *Drosophila* parasitoid *Leptopilina japonica*. Ecol Res. 2015 May 4;30(3):499–505.

49. Hood GR, Forbes AA, Powell THQ, Egan SP, Hamerlinck G, Smith JJ, et al. Sequential divergence and the multiplicative origin of community diversity. Proceedings of the National Academy of Sciences. 2015 Nov 3;112(44).

50. Forbes AA, Devine SN, Hippee AC, Tvedte ES, Ward AKG, Widmayer HA, et al. Revisiting the particular role of host shifts in initiating insect speciation. Evolution (N Y). 2017 May 16;71(5):1126–37.

51. Braga MP, Guimarães PR, Wheat CW, Nylin S, Janz N. Unifying host-associated diversification processes using butterfly–plant networks. Nat Commun. 2018 Dec 4;9(1):5155.

52. Ehrlich PR, Raven PH. Butterflies and plants: A study in coevolution. Evolution (N Y). 1964 Dec;18(4):586–608.

53. Janz N, Nylin S. The oscillation hypothesis of host-plant range and speciation. In: Tilmon K, editor. Specialization, Speciation, and Radiation: The Evolutionary Biology of Herbivorous Insects [Internet]. University of California Press; 2008. p. 203–15. Available from: https://academic.oup.com/california-scholarship-online/book/14621/chapter/168758186

54. Farrell MJ, Park AW, Cressler CE, Dallas T, Huang S, Mideo N, et al. The ghost of hosts past: impacts of host extinction on parasite specificity. Philosophical Transactions of the Royal Society B: Biological Sciences. 2021 Nov 8;376(1837):20200351.

Figure 2

Click here to access/download;Figure;Fig 2.tif

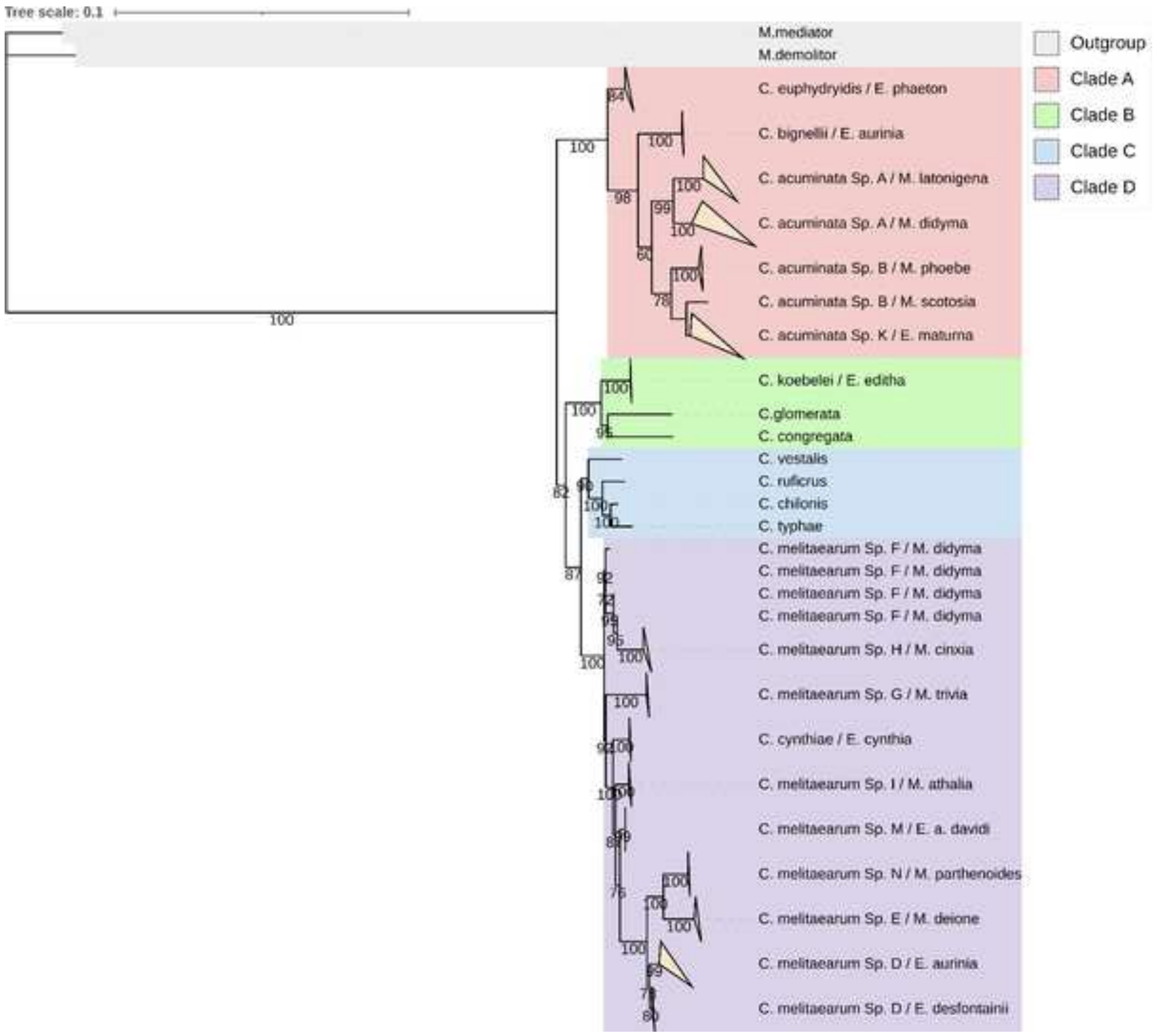

Figure 3

Click here to access/download;Figure;Fig 3.tif

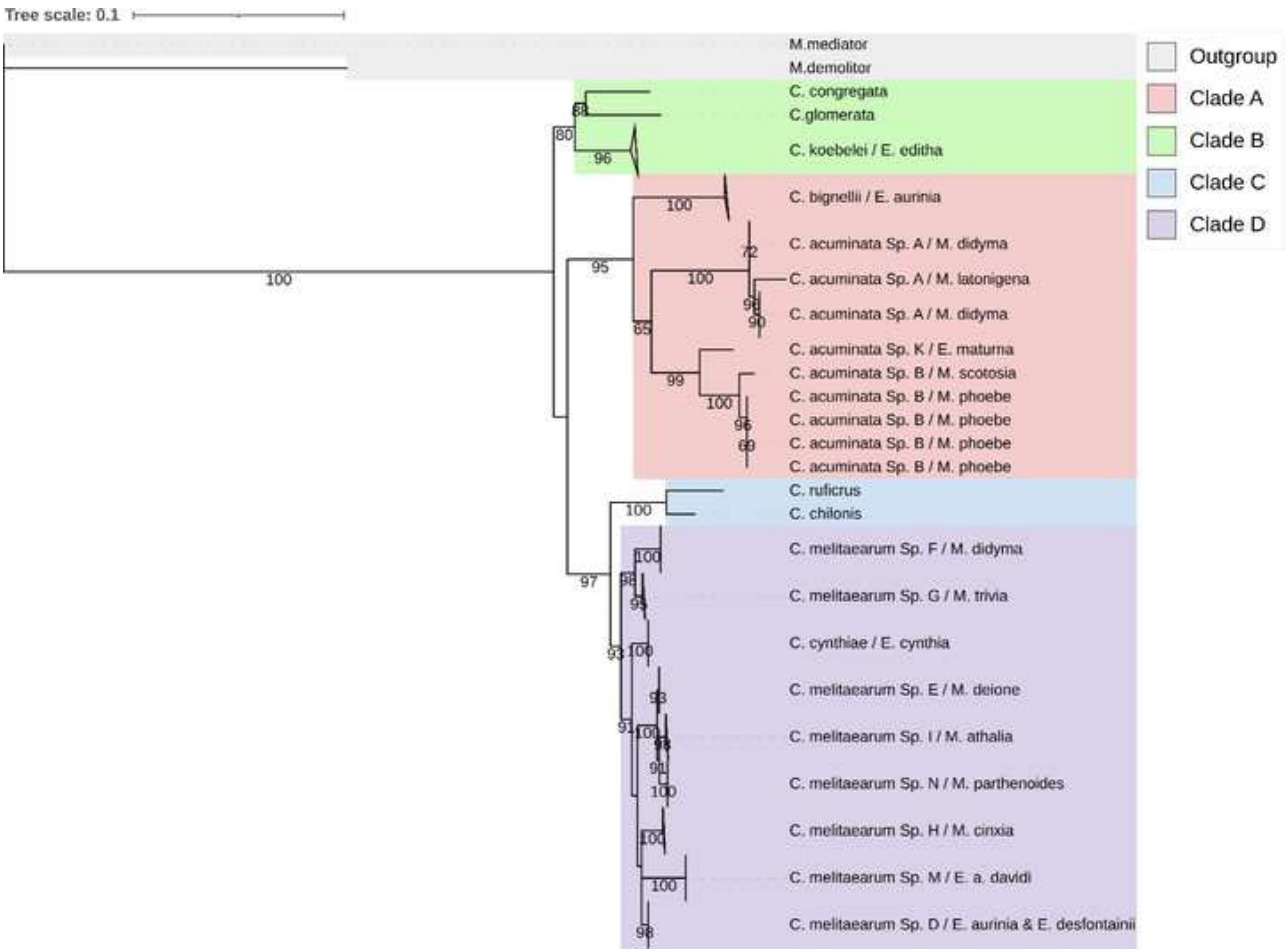

Figure 1

Click here to access/download;Figure;Fig 1.tif

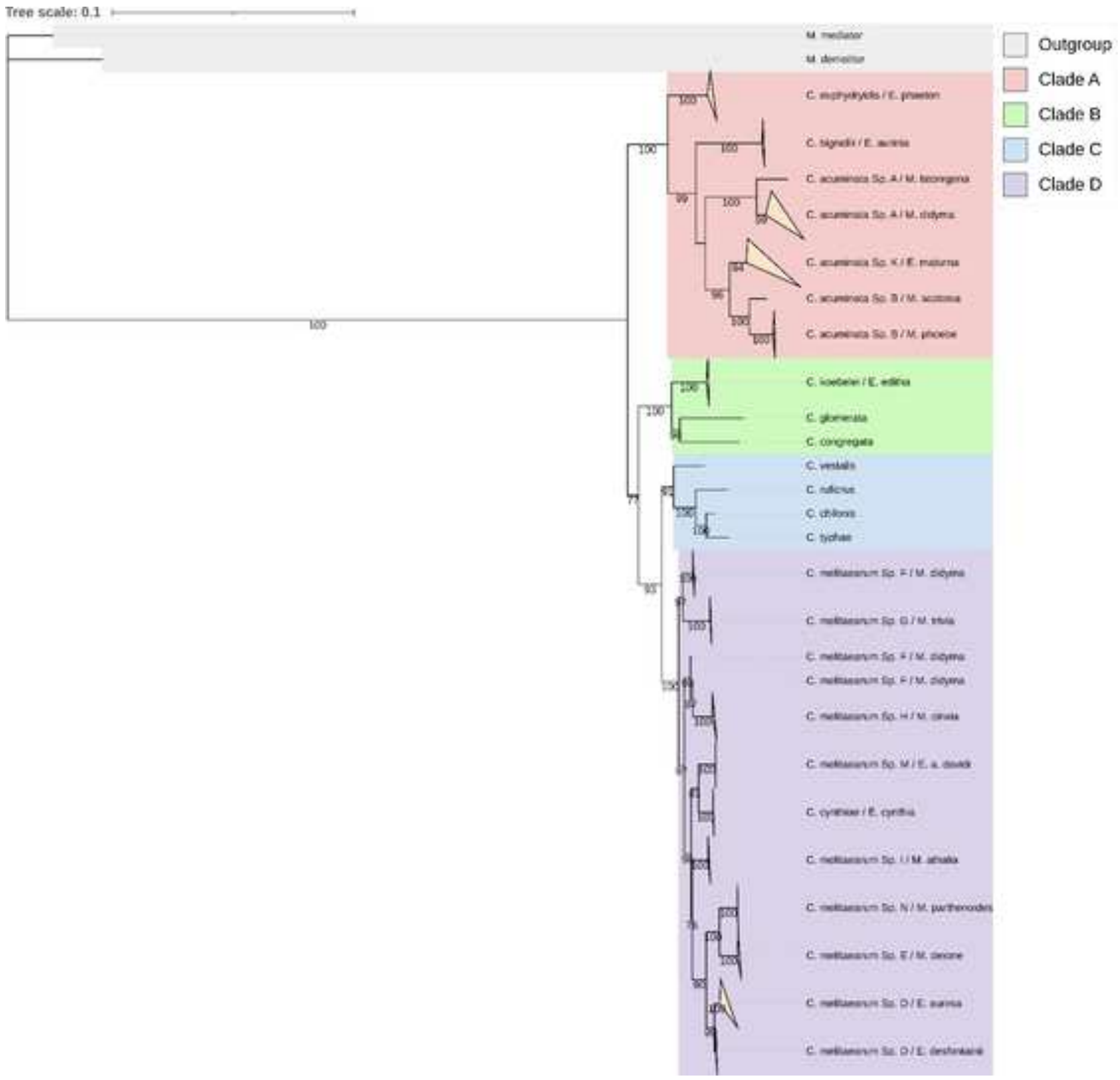

Click here to access/download
Supporting Information
SCHACH_Supplementary material Cotesia Phylogeny_6-1-2026.docx
