# Supplementary material for: Progress in the molecular phylogeny of Cotesia acuminata and C. melitaearum cryptic species complexes
Source: PLoS One. 2026 May 27;21(5):e0348973. doi: 10.1371/journal.pone.0348973 (PMC13215553; doi:10.1371/journal.pone.0348973)
Supplement: Attachment — Submitted filename: PONE-D-26-02522_reviewer_I.docx [file pone.0348973.s019.docx]

**PLOS One**

**Progress in the molecular phylogeny of Cotesiaacuminata and C. melitaearum cryptic species complexes**

#### --Manuscript Draft--

| **Manuscript Number:** | PONE-D-26-02522 |
| --- | --- |
| **Article Type:** | Research Article |
| **Full Title:** | Progress in the molecular phylogeny of Cotesiaacuminata and C. melitaearum cryptic species complexes |
| **Short Title:** | Progress in the molecular phylogeny of Cotesia acuminata and C. melitaearum cryptic species complexes |
| **Corresponding Author:** | Julian Schach  University of Helsinki: Helsingin Yliopisto Helsinki, FINLAND |
| **Keywords:** | Parasitoids, Maximum likelihood, Melitaeini, Systematics, Braconidae |
| **Abstract:** | Cryptic species present major challenges for biodiversity and evolutionary research due to the morphological similarity and frequent lack of available genetic and ecological data. In parasitoids wasps, this is especially true for lineages with limited sampling and unclear species boundaries. Here we reconstruct the phylogeny of Cotesia wasps parasitizing Melitaeini butterflies, including two cryptic species complexes (Cotesia acuminata agg. and C. melitaearum agg.). Using a ten-gene dataset from samples collected in Europe, Asia, and North America, we inferred relationships among 22 Cotesia species using maximum likelihood. We also included non-Melitaeini associated Cotesia to assess whether the parasitoids of Melitaeini form a monophyletic group. Our analyses yielded a highly supported phylogeny, revealing four major clades, three of which included the Melitaeini associated species. This confirms that the Cotesia parasitizing Melitaeini butterflies are polyphyletic, resulting from independent host shifts across the genus. Each clade is further subdivided into subclades corresponding to the different cryptic species complexes, clarifying previously unresolved relationships. These results provide a robust framework for future studies on the evolution, ecology, and host use dynamics of Cotesia wasps and highlight the utility of multi-locus data for resolving phylogenies in morphologically cryptic taxa. |
| **Order of Authors:** | Julian Schach |
|  | Federica Valerio |
|  | Anne Duplouy |
| **Additional Information:** |  |
| **Question** | **Response** |
| **Financial Disclosure**  Enter a financial disclosure statement that describes the sources of funding for the work included in this submission. Review the [submission guidelines](http://journals.plos.org/plosone/s/submission-guidelines#loc-financial-disclosure-statement) for detailed requirements. View published research articles from [*PLOS ONE*](https://journals.plos.org/plosone/) for specific examples.  This statement is required for submission and **will appear in the published article** if the submission is accepted. Please make | Yes |

| sure it is accurate. |  |
| --- | --- |
| **Funded studies**  Enter a statement with the following details:   - Initials of the authors who received each award - Grant numbers awarded to each author - The full name of each funder - URL of each funder website - Did the sponsors or funders play any role in the study design, data collection and analysis, decision to publish, or preparation of the manuscript? |  |
| Did you receive funding for this work? |  |
| Please add funding details.  as follow-up to "**Financial Disclosure**  Enter a financial disclosure statement that describes the sources of funding for the work included in this submission. Review the [submission guidelines](http://journals.plos.org/plosone/s/submission-guidelines#loc-financial-disclosure-statement) for detailed requirements. View published research articles from [*PLOS ONE*](https://journals.plos.org/plosone/) for specific examples.  This statement is required for submission and **will appear in the published article** if the submission is accepted. Please make sure it is accurate. | The work was funded by the Research Council of Finland to Dr. Anne Duplouy grant number 328944 |
| **Funded studies**  Enter a statement with the following details:   - Initials of the authors who received each award - Grant numbers awarded to each author - The full name of each funder - URL of each funder website - Did the sponsors or funders play any role in the study design, data collection and analysis, decision to publish, or preparation of the manuscript? |  |
| Did you receive funding for this work?" |  |
| Please select the country of your main research funder (please select carefully | FINLAND - FI |

| as in some cases this is used in fee calculation).  as follow-up to "**Financial Disclosure**  Enter a financial disclosure statement that describes the sources of funding for the work included in this submission. Review the [submission guidelines](http://journals.plos.org/plosone/s/submission-guidelines#loc-financial-disclosure-statement) for detailed requirements. View published research articles from [*PLOS ONE*](https://journals.plos.org/plosone/) for specific examples.  This statement is required for submission and **will appear in the published article** if the submission is accepted. Please make sure it is accurate. |  |
| --- | --- |
| **Funded studies**  Enter a statement with the following details:   - Initials of the authors who received each award - Grant numbers awarded to each author - The full name of each funder - URL of each funder website - Did the sponsors or funders play any role in the study design, data collection and analysis, decision to publish, or preparation of the manuscript? |  |
| Did you receive funding for this work?" |  |
| **Competing Interests**  Use the instructions below to enter a competing interest statement for this submission. On behalf of all authors, disclose any [competing interests](http://journals.plos.org/plosone/s/competing-interests) that could be perceived to bias this work—acknowledging all financial support and any other relevant financial or non- financial competing interests.  This statement is required for submission and **will appear in the published article** if the submission is accepted. Please make sure it is accurate and that any funding sources listed in your Funding Information later in the submission form are also | The authors have declared that no competing interests exist. |

| declared in your Financial Disclosure statement.  View published research articles from  [*PLOS ONE*](http://journals.plos.org/plosone/) for specific examples. |  |
| --- | --- |
| **NO authors have competing interests**  Enter: *The authors have declared that no competing interests exist.*  **Authors with competing interests**  Enter competing interest details beginning with this statement:  *I have read the journal's policy and the authors of this manuscript have the following competing interests: [insert competing interests here]* |  |
| * typeset |  |
| **Ethics Statement**  Enter an ethics statement for this submission. This statement is required if the study involved:   - Human participants - Human specimens or tissue - Vertebrate animals or cephalopods - Vertebrate embryos or tissues - Field research   Write "N/A" if the submission does not require an ethics statement.  General guidance is provided below. Consult the [submission guidelines](http://journals.plos.org/plosone/s/submission-guidelines#loc-guidelines-for-specific-study-types) for detailed instructions. **Make sure that all information entered here is included in the Methods section of the manuscript.** | N/A |

| **Format for specific study types**  **Human Subject Research (involving human participants and/or tissue)**   - Give the name of the institutional review board or ethics committee that approved the study - Include the approval number and/or a statement indicating approval of this research - Indicate the form of consent obtained (written/oral) or the reason that consent was not obtained (e.g. the data were analyzed anonymously)   **Animal Research (involving vertebrate animals, embryos or tissues)**   - Provide the name of the Institutional Animal Care and Use Committee (IACUC) or other relevant ethics board that reviewed the study protocol, and indicate whether they approved this research or granted a formal waiver of ethical approval - Include an approval number if one was obtained - If the study involved *non-human primates*, add *additional details* about animal welfare and steps taken to ameliorate suffering - If anesthesia, euthanasia, or any kind of animal sacrifice is part of the study, include briefly which substances and/or methods were applied   **Field Research**  Include the following details if this study involves the collection of plant, animal, or other materials from a natural setting:   - Field permit number - Name of the institution or relevant body that granted permission |  |
| --- | --- |
|  |  |
| **Data Availability**  Authors are required to make all data underlying the findings described fully available, without restriction, and from the time of publication. PLOS allows rare exceptions to address legal and ethical concerns. See the [PLOS Data Policy](http://journals.plos.org/plosone/s/data-availability) and [FAQ](http://journals.plos.org/plosone/s/data-availability#loc-faqs-for-data-policy) for detailed information. | Yes - all data are fully available without restriction |

| A Data Availability Statement describing where the data can be found is required at submission. Your answers to this question constitute the Data Availability Statement and **will be published in the article**, if accepted. |  |
| --- | --- |
| **Important:** Stating ‘data available on request from the author’ is not sufficient. If your data are only available upon request, select ‘No’ for the first question and explain your exceptional situation in the text box. |  |
| Do the authors confirm that all data underlying the findings described in their manuscript are fully available without restriction? |  |
| **Describe where the data may be found in full sentences. If you are copying our sample text, replace any instances of XXX with the appropriate details.**   - If the data are **held or will be held in a public repository**, include URLs, accession numbers or DOIs. If this information will only be available after acceptance, indicate this by ticking the box below. For example: *All XXX files are available from the XXX database (accession number(s) XXX, XXX.*). - If the data are all contained **within the manuscript and/or Supporting Information files**, enter the following: *All relevant data are within the manuscript and its Supporting Information files.* - If neither of these applies but you are able to provide **details of access elsewhere**, with or without limitations, please do so. For example:   *Data cannot be shared publicly because of [XXX]. Data are available from the XXX Institutional Data Access / Ethics Committee (contact via XXX) for researchers who meet the criteria for access to confidential data.*  *The data underlying the results presented in the study are available from (include the name of the third party* | All sequence data has been uploaded to NCBI, with accession numbers provided. The sequencing data will be made public upon manuscript publication. Accession numbers PX642706 - PX642796, PX648620 - PX649011 |

| *and contact information or URL).*   - This text is appropriate if the data are owned by a third party and authors do not have permission to share the data.   * typeset |  |
| --- | --- |
| Additional data availability information: | Tick here if the URLs/accession numbers/DOIs will be available only after acceptance of the manuscript for publication so that we can ensure their inclusion before publication. |

<https://www2.cloud.editorialmanager.com/pone/download.aspx?id=43342698&guid=7b67224e-5c41-4811-86b1-19c7054cd704&scheme=1>Manuscript	[Click here to access/download;Manuscript;Cotesia phylogeny manuscipt_14_1_2026.docx](https://www2.cloud.editorialmanager.com/pone/download.aspx?id=43342698&guid=7b67224e-5c41-4811-86b1-19c7054cd704&scheme=1)

# 1 Progress in the molecular phylogeny of

2	*Cotesia acuminata* and *C. melitaearum*

# 3 cryptic species complexes

4 Julian Schach^1^, Federica Valerio^1^, Anne Duplouy^1,2^
5 ^1^ Department of Organismal and Evolutionary Biology, Faculty of Biological and
6 Environmental Sciences, the University of Helsinki, Helsinki, Finland
7 2 Institute of Life Sciences (HiLIFE), the University of Helsinki, Helsinki, Finland
8 [Julian.schach@helsinki.fi](mailto:Julian.schach@helsinki.fi), [Federica.valerio@helsinki.fi](mailto:Federica.valerio@helsinki.fi), [anne.duplouy@helsinki.fi](mailto:anne.duplouy@helsinki.fi)
9 Corresponding Author – Julian Schach, [Julian.schach@helsinki.fi](mailto:Julian.schach@helsinki.fi), Viikinkaari 1, 00790 Helsinki

## 10 Author Contributions

11 JS – Investigation, data curation, methodology, analysis, writing – original draft, writing – review
12 and editing. FV – Methodology, supervision, writing – review and editing. AD –
13 Conceptualization, funding acquisition, project administration, supervision, writing – review and
14 editing.
15 **Keywords:** Parasitoids, Maximum likelihood, Melitaeini, Systematics, Braconidae 16

17

## 18 Abstract

19 Cryptic species present major challenges for biodiversity and evolutionary research due to the
20 morphological similarity and frequent lack of available genetic and ecological data. In
21 parasitoids wasps, this is especially true for lineages with limited sampling and unclear species
22 boundaries. Here we reconstruct the phylogeny of *Cotesia* wasps parasitizing Melitaeini
23 butterflies, including two cryptic species complexes (*Cotesia acuminata agg.* and *C.*
24 *melitaearum agg.*). Using a ten-gene dataset from samples collected in Europe, Asia, and North
25 America, we inferred relationships among 22 *Cotesia* species using maximum likelihood. We
26 also included non-Melitaeini associated *Cotesia* to assess whether the parasitoids of Melitaeini
27 form a monophyletic group. Our analyses yielded a highly supported phylogeny, revealing four
28 major clades, three of which included the Melitaeini associated species. This confirms that the
29 *Cotesia* parasitizing Melitaeini butterflies are polyphyletic, resulting from independent host shifts
30 across the genus. Each clade is further subdivided into subclades corresponding to the different
31 cryptic species complexes, clarifying previously unresolved relationships. These results provide
32 a robust framework for future studies on the evolution, ecology, and host use dynamics of
33 *Cotesia* wasps and highlight the utility of multi-locus data for resolving phylogenies in
34 morphologically cryptic taxa.

## 35 Introduction

36 Species have historically been described using morphological data, and species relationships
37 based on degrees of similarities of morphological characteristics. More recently, the advent of
38 molecular techniques has refined many taxonomic/phylogenetic classifications, by allowing us to
39 study species relationships and biodiversity with much higher resolution (1). Cryptic species, are
40 two or more molecularly distinct species, often accompanied by rapid speciation, which are or
41 have been erroneously classified as a single species because they are morphologically
42 indistinguishable (2). Such cryptic species are known to span the tree of life (3–6). Their hidden
43 genetic diversity has however challenged our ability to estimate the true biodiversity richness on
44 Earth, as many remain to be discovered and characterized (7). Furthermore, with the current
45 biodiversity crisis, we are likely losing many such species at alarming rates even before they
46 have been taxonomically characterized, or before their ecosystem roles were understood (8).
47 Parasitoid wasps make up one of the most diverse animal groups in the world (9). They lay their
48 eggs within or on their hosts’ body, and the parasitoid larvae develop feeding on the host, until
49 emergence of the adult wasp (10). These parasitoids can be very efficient at killing their hosts
50 and can act as key evolutionary pressures on their host species population growth (11) and host
51 population dynamics (12). For example, It has been shown that the specialist species *Cotesia*
52 *melitaearum* (Wilkinson), parasitizes only 10% of *Melitaea cinxia* (Linnaeus) caterpillars but can
53 still cause localized extinctions within the butterfly host metapopulation (12). Because of their narrow
54 host ranges and highly efficient killing of their hosts, several species of parasitoids have become
55 popular biocontrol agents in sustainable agriculture, contributing significantly to the $417 billion
56 USD global value of insect biocontrol (Brock et al. 2021). For example, *Cotesia ruficrus*
57 (Haliday), parasitizes the fall armyworm (*Spodoptera frugiperda,* Smith), which is contributing to
58 food insecurity in Africa, Asia, and the Pacific (13).
59 Most of the worlds parasitoid diversity occurs within the superfamily Ichneumonoidea, which
60 includes the two families Ichneumonidae (Latreille) and Braconidae (Nees von Esenbeck), with
61 approximately 60,000 described species and many more estimated (14). Within the
62 Braconidae, the subfamily Microgastrinae is the most diverse, with the genus *Cotesia*
63 (Cameron) alone including over 300 named species (Fernandez-Triana et al. 2020), and an
64 estimated 1500-2000 species worldwide (16). The *Cotesia* wasps usually exhibit narrow host
65 ranges, and closely related species often differ in their host species preferences, attacking one
66 or few closely related lepidopteran host species (17). For example, although previous studies
67 suggested that seven species of *Cotesia* were generalist parasites of the Melitaeini (Newman)
68 butterfly species found across Europe and Asia (18,19), more recent molecular analyses
69 accompanied by rearing experiments provided evidence that the originally considered generalist
70 parasitoid species, *Cotesia acuminata* (Reinhard), and *C. melitaearum*, were in fact comprised of numerous
71 cryptic species, each with narrow Melitaeini butterfly host specificity (17,20).
72 In the complex species community of Melitaeini butterflies occurring in Catalonia in northeastern
73 Spain (Catalan Butterfly Monitoring Scheme, CMBS), 10 local Melitaeini butterfly species were
74 identified as hosts to at least 10 *Cotesia* (cryptic) species (17). Unfortunately, the
75 original barriers to geneflow between the current cryptic lineages of the wasps remain unclear,
76 as studies exploring isolation by distance or endosymbiotic bacteria failed to explain the
77 sympatric speciation of the parasitoid species (17,21,22). Currently, the phylogeny of the
78 Catalonian *C. acuminata* complex and *C. melitaearum* complex are based on few microsatellite loci
79 (17), while a wider Eurasian phylogeny of these groups combined two mitochondrial (COI and ND1)
80 and one nuclear (ITS2) gene sequences (18). As a consequence of the limited
81 available genetic data included in these phylogenies, the relationships between *Cotesia*
82 species still contain several unresolved nodes (i.e., nodes with low bootstrap support estimates;
83 Kankare and Shaw 2004, Kankare et al. 2005b), and we still lack a clear description of the
84 diversity of and the speciation scenarios between *Cotesia* wasps attacking Melitaeini butterflies.
85 Here, we aimed to refine the phylogeny of *Cotesia* species associated with 16 species of
86 Melitaeini butterflies, by using a larger number of nuclear markers and a wider sample diversity
87 than previous studies. We analysed sequences of two mtDNA and eight nDNA markers from
88 both freshly collected specimens from Finland, Italy, Spain, and Switzerland, as well as DNA
89 extracts from samples collected between 1979-2002 from China, Finland, France, Hungary,
90 Russia, Spain, Sweden, and USA (18). With this, we expected to resolve the previously unclear
91 nodes in the *Cotesia* phylogeny, particularly the basal nodes, and to provide a strong
92 phylogenetic reconstruction within the cryptic species complexes.

93

## 94 Materials and Methods

### 95 Samples

96 This study includes *Cotesia* parasitoid wasp specimens that had emerged from the caterpillars
97 of 16 species of Melitaeini butterflies. These Include *Euphydryas aurinia* (Rottemburg) *Euphydryas*
98 *aurinia davidi* (Wahlurg)*, Euphydryas cynthia* (Dennis and Schiffermueller)*, Euphydryas*
99 *desfontainii* (Godart)*, Euphydryas editha* (Boisduval)*, Euphydryas maturna* (Linnaeus)*,*
100 *Euphydryas phaeton* (Drury)*, Melitaea athalia* (Rottemburg)*, Melitaea cinxia, Melitaea deione*
101 (Geyer)*, Melitaea didyma* (Esper)*, Melitaea latonigena* (Eversmann)*, Melitaea parthenoides*
102 (Keferstein)*, Melitaea phoebe* (Dennis and Schiffermueller)*, Melitaea scotosia* (Butler)*,* and
103 *Melitaea trivia* (Dennis and Schiffermueller) (Table 1).
104 Among the *Cotesia* wasps*,* two cryptic species complexes are known to consist of multiple
105 species: *C. acuminata* and *C. melitaearum*. Following previously introduced convention (20), we
106 refer to these as *C. acuminata agg.* and *C. melitaearum agg.,* with individual lineages denoted
107 by species-level designations (e.g. *Sp. A., Sp. B.*)
108 The Melitaeini caterpillars were collected over two time periods: 1979-2002 and 2022-2023. The
109 DNA from early samples collected for the study of *Cotesia* wasps’ phylogeny and butterfly-host
110 specialization in earlier studies was extracted in the early 2000’s and preserved in the -20 °C
111 freezers at the University of Helsinki (17,18,20). These DNA extracts represent 10 species from
112 eight different countries, including China (N=3 from 1 species), Finland (N=5 from 1 species),
113 France (N=7 from 3 species), Hungary (N=3 from 1 species), Russia (N=6 from 2 species),
114 Spain (N=2 from 1 species), Sweden (N=3 from 1 species), and USA (N=14 from 2 species)
115 (Table 1). Additionally, caterpillars from one locality in Finland (Åland) were also collected in the
116 fall of 2022. Finally, parasitized Melitaeini caterpillars were collected in early April 2023 from five
117 localities in Catalonia, Spain (Coll d'Estenalles, El Brull, Fígols-El Forn, Pyrenees), two localities
118 in Switzerland (Vaud and Valais), and one locality in Italy (Aosta). After collection, the
119 caterpillars were reared in the laboratory on excess of their respective host plant, until the adult
120 wasps emerged. Freshly emerged adult wasps were killed by placing them in a freezer for 24
121 hours. The samples were stored in 90% ethanol at -20℃ until further manipulated.
122 Table 1. *Cotesia* identity, host species, number of individuals, caterpillar host plant, country,
123 locality, collector ID, and finally collection year. “-” indicates the information was unavailable.

***Cotesia* species**

**Butterfly host species**

**# of Ind.**

**Identified host plant**

**Country**

**Locality**

**Collector	Collection**

**year**

*C. acuminata Sp. A	Melitaea*

*latonigena*

3	-	Russia	Siberia	N.W. I.H.	1998

*C. acuminata Sp. A	Melitaea*

*didyma*

_5_	*Plantago lanceolata*

Spain	Coll d'Estenalles

F.V.	2023

*C. acuminata Sp. A	Melitaea*

*didyma*

*C. acuminata Sp. B	Melitaea*

*scotosia*

*C. acuminata Sp. B	Melitaea*

*phoebe*

*Euphydryas*

5	-	Switzerland	Conthey	Y.C.	2023

3	-	China	Peking	I.H., L.G.	1999

5	-	Italy	Aosta	Y.C.	2023

*C. acuminata Sp. K*

*maturna*

*C. acuminata Sp. K	Euphydryas*

*maturna*

*Melitaea*

*C. acuminata Sp. L*

*athalia*

*C. bignellii	Euphydryas aurinia*

*Euphydryas*

*C. cynthiae*

*C. euphydryidis*

*C. koebelei*

*C. melitaearum Sp. D*

*cynthia*

*Euphydryas phaeton*

*Euphydryas editha*

*Euphydryas desfontainii*

_5_	*Cephalaria leucantha*

| 1 | France,  - | | Cote d'Or, Malloy, | P.J.C.R. | 1999 |
| --- | --- | --- | --- | --- | --- |
|  |  | | Lindesberg |  |  |
| 3 | - Sweden | | Lindesberg | C.U.E. | 1999 |
| 1 | - France | | Var, St. Paul-en- | P.W.C. | 1982 |
|  |  | | Foret |  |  |
| 5 | - | Switzerland | La Vraconnaz | Y.C. | 2023 |
| 4 | - France | | Alps, Laus de | M.S. | 2001 |
|  |  | | Cervieres |  |  |
| 7 | - USA | | Warren Co., Front | N.S. | 1979 |
|  |  | | Royal |  |  |
| 7 | - USA | | Yosemite National | M.S., B.W. | 2001 |
|  |  | | Park |  |  |

Spain	Fígols-El Forn

CS	2023

##### species

| **Butterfly # of Identified**  ***Cotesia* species host Ind. host plant Country** | **Locality** | **Collector** | **Collection year** |
| --- | --- | --- | --- |
| *C. melitaearum Sp. Euphydryas Lonicera*  *D aurinia* ^5^ *implexa* Spain | Cornudella  de | C.S. | 2023 |
|  | Montsant |  |  |
| *C. melitaearum Sp. Euphydryas* _5_ *Lonicera* Spain | La Mussara | C.S. | 2023 |
| *C. melitaearum Sp. Melitaea* _5_ *Antirhinum* Spain | Les Illes | CS | 2023 |
| *C. melitaearum Sp. Melitaea* _5_ *Antirhinum* Spain | Poboleda | CS | 2023 |
| *C. melitaearum Sp. Melitaea* 3 - Hungary | Örseg | M.R.S. | 2001 |
| *C. melitaearum Sp. Melitaea* 2 - Spain | Cantallops | C.S. | 2002 |
| *C. melitaearum Sp. Melitaea* _10_ *Verbascum* Spain | Pyrenees | C.S. | 2023 |
| *C. melitaearum Sp. Melitaea* _8_ *Plantago* Finland | Åland | S.I. | 2022 |
| *C. melitaearum Sp. Melitaea* 5 - Finland | Åland | L.G., S.vN. | 1997 |
| *C. melitaearum Sp. Euphydryas*  *M aurinia* 3 - Russia | Siberia | N.W., I.H. | 1998 |
| *davidi* |  |  |  |
| *C. melitaearum Sp. Melitaea* _5_ *Plantago* Spain  *N parthenoides media* | El Brull | C.S. | 2023 |

D *aurinia	implexa*
E *deione	majus*
E *deione	majus*
F *didyma*
F *didyma*
G *trivia	pulverulentum*
H *cinxia	lanceolata*
I *athalia*
124 *Collectors:* Y.C., Yannick Chitarro; P.W.C, Peter W. Cribb; C.U.E., Clae U. Eliasson; L.G., Lei
125 Guanchung; I.H., Ilkka Hanski; S.I., Suvi Ikonen: P.J.C.R., Peter J. C. Russel: M.R.S., Mark R.
126 Shaw; M.S., Michael Singer; N.S., Nancy Stamp; C.S., Constanti Stefansescu; S. vN., Saskya
127 van Nouhys; N.W. Niklas Wahlberg; B.W., Brian Wee; F.V. Federica Valerio
128 To test whether the *Cotesia* species parasitizing Melitaeini butterflies form a monophyletic
129 group, we also included gene sequences retrieved from *Cotesia chilonis* (Munakata)*, C.*
130 *congregata* (Say)*, C. glomerata* (Linnaeus)*, C. ruficrus, C. typhae* (Fernández-
131 Triana)*,* and *C. vestalis* (Haliday)*,* for which full genomes are publicly available from the NCBI
132 database. These *Cotesia* species naturally parasitize pest species of moths (e.g. *Spodoptera*
133 (Guenée), or *Manduca* (Hübner)) or butterflies (e.g. *Pieris* (Schrank)) (23,24). We also included
134 respective gene sequences from two Microgastrine species (*Microplitis demolitor* (Wilkinson)*,*
135 and *M. mediator* (Haliday)), for which full genomes are publicly available from the NCBI
136 database (Table 2), to use as outgroup in the phylogenetic analyses described below. Our sequences
137 were blasted against the respective genomes to extract the sequences of the genes used in the
138 phylogeny. All NCBI references can be found in Table 2.
139 Table 2. Host species, GenBank accession number, and reference for seven species added to
140 the *Cotesia* ingroup and *Microplitis* outgroup of the phylogenetic tree. “-“ indicates the
141 information was unavailable.

**Cotesia Species**

**Host**

**GenBank accession #**

**Reference**

*Cotesia chilonis	Chilo suppresalis*	GCA_018835575.1	(25) *Cotesia congregata	Manduca sexta*	GCA_905319865.3	(26) *Cotesia glomerata	-*	GCA_020080835.1	(27)

*Cotesia ruficrus	Mythimna separata*	GCA_048537615.1	- *Cotesia typhae	Sesamia nonagrioides*	GCA_013202065.2	(28) *Cotesia vestalis	Plutella xylostella*	GCA_000956155.1	-

*Microplitis demolitor	Pseudoplusia includens*	GCA_026212275.2	(29) *Microplitis mediator	-*	GCA_029852145.1	- 142

143 Depending on availability, we selected one to seven (on average five) parasitoid specimens per
144 caterpillar species, and per locality sampled. As expected, the cryptic nature of the parasitoids,
145 and the fact that some caterpillar species can host multiple species of parasitoids makes their
146 identification difficult using only morphology. The species identity of the parasitoids collected in
147 the early 2000’s was previously confirmed through morphology, *COI,* and host associations
148 (18). For the samples collected in 2022 and 2023, species identities were assigned after
149 blasting their respective *COI* sequences against the NCBI database, and by considering their
150 host associations, following previously established protocol (18).

151

### 152 DNA Extraction

153 For the samples collected in the early 2000’s the DNA was extracted using the NucleoSpin
154 Tissue Kit (Macherey-Nagel) following the manufacturers protocol and eluted in 50 μL of MilliQ
155 water (18). The samples have since been stored in the freezer (-20℃) at the University of
156 Helsinki, Finland. For the samples collected in 2022 and 2023, the DNA was extracted from
157 each whole insect, except in two cases where we pooled adult wasps that emerged from the
158 same host caterpillar. The Macherey-Nagel NucleoSpin Tissue Kit (Düren, Germany) was used
159 following the manufacturer’s protocol. The DNA was eluted twice in 40 μL of elution buffer to
160 increase final yield and concentration.

161

### 162 Gene selection

163 Genetic markers and primer pairs designed and tested by previous studies were selected
164 by reviewing the available literature on *Cotesia* and Braconidae phylogenies (See Table 2. for
165 references). All primer pairs were tested for specificity to *Cotesia* using Primer-Blast (30).
166 Additionally, 16 BUSCO genes were randomly selected from the *C. congregata* genome
167 (GCA_905319865.3) using BUSCO v 5.7.1 (31). Using Primer-Blast (30), we designed an
168 additional set of 16 primer pairs to amplify these 16 BUSCO genes. The best pairs were
169 selected based on the lowest self-complimentary score given by Primer-Blast. In total, two
170 mtDNA markers and 23 nDNA markers were tested, but only two and eight, respectively, were
171 successfully used in this study, as 13 other nDNA primer pairs designed failed to amplify
172 through gradient PCR (Table S5). All primer pairs, from the literature and designed for this
173 study, targeted genetic regions with a length ranging from 150 to 850 bp (Table 3).

175

176 Table 3. Characteristics of the primer pairs used in this study. Including the name of the marker
177 targeted (gene), primer name, primer sequence, amplicon size, annealing temperature, and
178 source. “-” indicates the information was unavailable.

| **Gene** | **Primer name** | **Sequence (5'-3')** | **Amplico size** | **n Ta Primer source (℃)** |
| --- | --- | --- | --- | --- |
| *COI* | LCO2198 | 5`- GGTCAACAAATCATAAAGATATTGG | 658 | 49 Folmer et al., 1994 |
|  | HCO1490 | 5`- TAAACTTCAGGGTGACCAAAAAATCA |  |  |
| *16s* | 16SWb | 5`- CACCTGTTTATCAAAAACAT | 375 | _58_ Dowton and Austin, 1994; Whitfield, 1997 |
|  | 16S outer | 5`- CTTATTCAACATCGAGGTC |  |  |
| *18s* | 18s - F | 5`- CATTGGAGGGCAAGTCTGGTGCCA | 240 | 58 Quicke et al. 2020 |
|  | 18s - R | 5`- AGTAAACGTACCGGCCCTCCTCG |  |  |
| *n28s rDNA* | 28s | 5`- AAGAGAGAGTTCAAGAGTACGTG | 618 | _56_ Mardulyn & Whitfield, 1999 |
|  | 28s-PM | 5`- TAGTTCACCATCTTTCGGGTCCC |  |  |
| *EF1a* | EF1A1 - F | 5`- AGATGGGYAARGGTTCCTTCAA | 418 | _56_ Belshaw and Quike, 1997 |
|  | EF1A1 - R | 5`- AACATGTTGTCDCCGTGCCATCC |  |  |
| *ITS2* | NG02955 | 5`- ATGAACATCGACATTTCGAACGCACA | - | 60 Regueira et al. 2017 |
|  | AB052895 | 5`- TTCTTTTCCTCCGCTTAGTAATATGCTTAA |  |  |
| *LW-Rh* | OpsFor2 | 5`-GGATGTASCTCCATTTGGTC | - | _58_ Banks and Whitfield, 2006 |
|  | Ops3'2 | 5`-AVHGATGCRACRTTCATTTTCT |  |  |
| *Elob* | TCEB - F | 5`-TCTTAAAAATTCCTCCAACGCATC | 250 | 57 This study |
|  | TCEB - R | 5`-GATGCCTGCTCTTGTCCATT |  |  |
| *SLD5* | SLD5 - F | 5`-CCTAAGAACGCGGTTGGAGA | 327 | 57 This study |
|  | SLD5 - R | 5`-TGAACAATATGCTGCGAGCC |  |  |

*Innexin*	INX-F	5`-CGCTCTTTGTGTGCTTGCTT	342	54	This study

INX-R	5`-GGTACAGAAGTCGGTGAGGC

179

### 180 PCR

181 Each 10 μL PCR reaction included 1 μL of Buffer solution, 0.8 μL of DNTPs (25nM) mix, 0.5 μL
182 of forward primers (10nM), 0.5 μL of reverse primers (10nM), 0.5 μL Mg2 reagent, 0.05 μL
183 KAPA Taq polymerase, 6.7 μL of H20 (sterile), and 1 μL of DNA extract. The PCR reactions
184 were initiated by incubation at 95°C for 3 min, followed by 30 cycles of 95°C for 40 s, the optimal
185 annealing temperature of the respective primer pair (Table 2) for 40 s, and 72°C for 40 s. The
186 PCR reaction ended with a final elongation step at 72°C for 5 min, and a final hold at 18°C.
187 The PCR products were run through a 1% agarose gel electrophoresis, with SYBR-safe stain
188 (Thermo Scientific, USA), and visualized under UV light. For each gel, 5 μL of 100 bp DNA
189 ladder (#SM0321, Thermo Scientific, USA) was loaded into the first well, and each subsequent
190 well was loaded with a mixture of 4 μL of both loading dye (bromophenol blue) and PCR
191 product. The gels were run for 30 minutes at 100 volts. The PCR amplification was repeated
192 once for any failed sample, to identify potential false negative results.

193

### 194 Preparation for Sanger Sequencing

195 The successfully amplified PCR products were individually cleaned using Exonuclease I (ExoI)
196 to remove any single-stranded DNA (Thermo Scientific, USA). For each reaction 0.5 μL of ExoI
197 was added to 5 μL of PCR product, then the samples were incubated at 37°C for 15 minutes,
198 followed by 15 minutes at 85°C to terminate the reaction.
199 Cleaned PCR products were sent to Macrogen Europe for Sanger Sequencing using the
200 company Multiple Primer/Plate option.

201

### 202 Sequence Cleaning and Alignments

203 The chromatograms of each sequence were manually quality-checked by using Geneious Prime
204 2025.01 ([https://www.geneious.com](https://www.geneious.com/)). Low-quality sequences (error probability limit 0.05) and
205 highly incomplete sequences (less than half of target length) were removed from further
206 analysis.
207 We included the respective gene sequences from two Microgastrine species (*Microplitis*
208 *demolitor* and *M. mediator*) as outgroup in the phylogenies described below.
209 Additionally, six species of *Cotesia* that do not parasitize Melitaeini were added to the ingroup
210 (Table 2). For each maker, all sequences were aligned with MUSCLE (32), and all alignments
211 were manually curated with AliView (33). The accession numbers for the sequences submitted
212 to NCBI are PX523132-PX523219, PX559195-PX559573, PX642706-PX642796, and
213 PX648620-PX649011.
214 *Cotesia* Phylogeny
215 For the phylogenetic analysis we used maximum likelihood (ML) with IQ-TREE V2.4.0 (34).
216 Phylogenetic analyses were conducted for: (I) all ten genes concatenated (mtDNA and nDNA),
217 (II) eight concatenated nuclear genes, (III) two concatenated mitochondrial genes, and (IV) all
218 genes independently. Specimens that amplified for less than half of the markers were excluded
219 from concatenated analysis but kept for the individual gene tree analyses. Phylogenetic trees
220 were visualized using ITOL (35).
221 For ML analysis, we used ModelFinder (36), and the Bayesian Information Criterion (BIC) to
222 select for the best models. The partition finding algorithm (37) was run for the concatenated
223 data (ten-genes, nDNA, mtDNA) partitioned by genes using the MFP+MERGE option. Support
224 for nodes was evaluated with 1000 ultrafast bootstrap (UFBoot) approximations (38).

## 225 Results

### 226 Parasitoid Identification

227 The N=42 specimens collected from 1979 to 2002 include 10 species of *Cotesia*: four cryptic
228 species of *C. acuminata agg*, *C. cynthiae* Nixon 1974*, C. euphydryidis, C.*
229 *koebelei* Riley, 1889*,* and three cryptic species of *C. melitaearum*, either as previously identified
230 by earlier studies (17,18) or by comparing their COI sequences to NCBI*.* The sequencing results
231 of the 2022 and 2023 collections confirmed that these N=68 specimens include eight species of
232 *Cotesia*: *C. bignellii* Marshall, 1885*,* two cryptic species of *C. acuminata agg.,* and five
233 cryptic species of *C. melitaearum agg.*

### 234 Sequencing results

235 Of the 110 specimens sequenced for this study, 41% were successfully sequenced for all 10 genes,
236 66% for at least 9 genes, 75% for at least 8 genes, 85% for at least 7 genes, 88% for at least 6
237 genes, and 90% for at least 5 genes.

238

239 *Cotesia* Phylogenies

#### 240 (I) Ten-gene dataset

241 The ten-gene dataset included 110 wasp specimens (102 Melitaeini associated, and eight from
242 NCBI). The ten-gene alignment length was 3486 bp long, of which 1498 sites (43%)
243 represented unique site-patterns. Out of these, 747 sites (21% of all sites) were parsimony-
244 informative. The model partitioning algorithm revealed five partitions in the ten-gene dataset
245 (Table S1.)
246 The ML analysis revealed four major clades with high support (bootstrap = 100, 77, 93) and
247 suggested that the species attacking Melitaeini butterflies are polyphyletic (Fig. 1.).
248 •	**Clade A** contains the *C. acuminata agg., C. bignellii,* and *C. euphydryidis* parasitoids
249 from Melitaeini butterflies*. Cotesia euphydryidis* is placed at the base of the clade. The
250 next division is between *C. bignellii* and *C. acuminata agg.* The clade reveals that *C.*
251 *acuminata agg.* is monophyletic, where *Sp. B* and *K* cluster together and are sister taxa
252 to *Sp. A.* The divergence between the *Sp. A*, and *B/K* is poorly supported (bootstrap =
253 49), but the divergence between *Sp. B* and *K* is well supported.
254 •	**Clade B** contains *C. koebelei, C. congregata* and *C. glomerata* parasitoid wasps.
255 *Cotesia congregata* and *C. glomerata* do not use Melitaeini butterflies as hosts, but *C.*
256 *koebelei* wasps parasitize the Melitaeini species *E. editha.* The nodes in this clade are
257 highly supported (bootstrap = 98 and above).
258 •	**Clade C** contains *C. chilonis, C. ruficrus, C. typhae,* and *C. vestalis* parasitoids, which all
259 parasitize non-Melitaeini hosts. The divergence between these species is highly
260 supported (bootstrap = 93 and above).
261 •	**Clade D** contains *C. cynthiae* and *C. melitaearum agg,* which parasitize Melitaeini
262 butterflies*.* Ten subclades are revealed, represented by the individual cryptic species
263 and *C. cynthiae* with moderate to high support (bootstrap 77 to 100). Here, *C.*
264 *melitaearum agg.* forms a paraphyletic group. Most of the individual species within this
265 clade group together, forming monophyletic groups, except *C. melitaearum Sp. F*. For
266 this particular species, our phylogeny places the specimens from Spain in a sister group
267 to *C. melitaearum Sp. G* also from Spain and the specimens from Hungary are in a
268 sister-group to the *C. melitaearum Sp. H* from Finland.
269 Figure 1. Maximum likelihood phylogeny of *Cotesia* species parasitizing different Melitaeini host
270 species and their relatives based on the ten concatenated mitochondrial and nuclear genes
271 (16s, *18s, 28s, COI, EF1a, INX, ITS2, LW-Rh, SLD5,* and *Elob*)*.* The specimens are labelled by
272 the names of the *Cotesia* species and Melitaeini host caterpillar species. Bootstrap support
273 values (1000 replicates) are indicated for supported branches (≥60). Colors represent the main
274 clades (A, B, C, D). Branches corresponding to the same species were collapsed for visual
275 clarity and represented by triangles.

#### 276 (II) Nuclear-gene dataset

277 The nuclear-gene dataset included 112 wasp specimens (104 Melitaeini-associated, and eight
278 from NCBI). The concatenated alignment was 2413 bp long, of which 1035 sites (43%)
279 represented unique site-patterns, and of these 494 (20% of all sites) were parsimony
280 informative. The model partitioning algorithm revealed five partitions in the nuclear-gene dataset
281 (Table S2).
282 The ML analysis produced a highly supported phylogeny that is largely congruent with the ten-
283 gene tree. The same four major clades were recovered with strong support (bootstrap = 100,
284 82, 87) with key differences being the topology within clades A and D (Fig. 2.).
285 •	**Clade A** now groups *C. acuminata Sp. B* emerging from *M. scotosia* with *C. acuminata*
286 *Sp. K.* Again, the relationships between the cryptic species have lower support
287 (bootstrap = 60).
288 •	**Clade D** shows some discordance from the ten-gene phylogeny. For example, *C.*
289 *melitaearum Sp. F* is grouped together with *C. melitaearum Sp. H* and is no longer
290 polyphyletic, thus resulting in nine subclades. Additionally, *C. cynthiae* and *C.*
291 *melitaearum Sp. M* are no longer sister groups. Meanwhile, the relationships between *C.*
292 *melitaearum Sp. N, E* and *D* are concordant with the ten-gene dataset.
293 Figure 2. Maximum likelihood phylogeny of *Cotesia* species parasitizing different Melitaeini host
294 species and their relatives based on the nuclear genes (*18s, 28s, EF1a, INX, ITS2, LW-Rh,*
295 *SLD5,* and *Elob*)*.* The specimens are labelled by the names of the *Cotesia* species and
296 Melitaeini host caterpillar species. Branches corresponding to the same species were collapsed
297 for visual clarity and represented by triangles. Bootstrap support values (1000 replicates) are
298 indicated for supported branches (≥60). Colors represent the main clades (A, B, C, D).

#### 299 (III) Mitochondrial-gene dataset

300 The mitochondrial-gene dataset included the sequences of the *16s* and *COI* genes from 92
301 specimens (86 Melitaeini associated, six from NCBI). The mitochondrial alignment was 1072bp
302 long, of which 451 (42%) represented unique site-patterns, of which 242 sites (23% of all sites)
303 were parsimony informative. The model partitioning algorithm revealed two partitions in the
304 mitochondrial gene dataset, which correspond to the two markers (Table S3). No specimens
305 from *C. euphydryidis* and *C. melitaearum Sp. F* from Hungary amplified for either mitochondrial
306 marker, and no sequences were found from *C. typhae* and *C. vestalis* genomic projects, thus
307 they are missing from the mitochondrial phylogenetic analysis.
308 The ML analysis revealed the same four major clades as the ten-gene and nuclear gene
309 phylogenies, however with varying support (bootstrap = 100, 49, 97) and topology.
310 •	**Clade B** now forms the basal clade of the phylogeny. The relationships within the clade
311 exposed in the ten-gene and nuclear phylogenies are conserved.
312 •	**Clade A** with **Clade C** and **Clade D** now group together as sister taxa to Clade B**.**
313 Clades C and D are sister clades to Clade A, which is congruent the ten-gene
314 phylogeny. The relationships within Clade A and C are congruent to the ten-gene
315 phylogeny, despite missing species. The relationships between *C. acuminata agg.*
316 species is low (bootstrap = 60). Within clade D, we can still observe the paraphyletic
317 nature of this cryptic complex, forming nine subclades comprised of the *C. melitaearum*
318 *agg.* and *C. cynthiae*. Additionally, *C. melitaearum Sp. D* does not cluster by the two
319 hosts. Many of the relationships in this clade are unsupported (bootstrap < 60) (Fig. 3.).

320

321 Figure 3. Maximum likelihood phylogeny of *Cotesia* species parasitizing different Melitaeini host
322 species and their relatives based on the mitochondrial genes (*16s* and *COI*). The specimens are
323 labelled by the names of the *Cotesia* species and Melitaeini host caterpillar species. Branches
324 corresponding to the same species were collapsed for visual clarity and represented by
325 triangles*.* Bootstrap support values (1000 replicates) are indicated for supported branches
326 (≥60). Colors represent the main clades (A, B, C, D).

#### 327 (IV) Individual Genes

328 All genes were also analysed individually using ML to identify discordance or outlier genes. The
329 substitution models for the ML analyses are listed in Table S4. Rather expectedly, the individual
330 gene phylogenies had much lower branch supports than either of the other phylogenies, likely
331 because of the small nucleotide sample size included in each analysis. Alone, the individual
332 gene trees are not phylogenetically informative. The major clades were only recovered in the
333 *16s*, *COI*, and *SLD5* ML trees, but the relationships within the clades, particularly within the
334 Clade C, remain incongruent (Figs. S1-S10.).

## 335 Discussion

336 Resolving species phylogenies is essential for studying trait evolution, diversification, and
337 ecological interactions, but can remain challenging in non-model cryptic species due to limited
338 access to sampling and molecular resources. By designing new primers and increasing the
339 number of available molecular markers from two to ten, we were able to produce the most
340 robust phylogeny of the *Cotesia* species parasitizing Melitaeini butterflies to date. This improved
341 phylogeny informs on previously hidden species diversity within this genus and is an important
342 resource for future research on Braconidae population genetics, evolutionary dynamics and
343 ecology.
344 Our analyses revealed that all specimens group in four highly supported clades (A-D), each
345 subdivided into distinct subclades of (cryptic) species. By including genetic sequences from
346 *Cotesia* species that do not attack Melitaeini butterflies, we confirm that the Melitaeini-
347 specialized species are polyphyletic, appearing in three of the four main *Cotesia* clades. This
348 suggests that *Cotesia* wasps have shifted hosts multiple times throughout their evolutionary
349 history. Such patterns of host shifts have been observed in other parasitoid lineages, including
350 Ichneumonoidea (39), and parasitoids of leaf miners (40), indicating that parasitoid evolution
351 often involves ecological switching rather than strict co-divergence with hosts.
352 Many of these Melitaeini-associated *Cotesia* species occur in sympatry, yet the drivers of
353 radiation and barriers to gene flow remain unclear (17,21). Host evolution appears to play a
354 partial role. For example, in Clade A, the basal species parasitize *Euphydryas* caterpillars, while
355 most *C. acuminata* species have shifted to *Melitaea.* Given that *Euphydryas* butterflies diverged
356 before *Melitaea* (41), it likely supports the hypothesis that the ancestral host for Clade A
357 belonged to the *Euphydryas* butterfly lineage. A similar pattern may exist in Clade B where *C.*
358 *koebelei* parasitizes *E. editha* and has also been tentatively reported on *Melitaea* (42). If *C.*
359 *koebelei* parasitizes both *Euphydryas* and *Melitaeini* butterflies, it could represent a cryptic
360 species complex, or a rare generalist able to attack both butterfly genera. The question of
361 which of these two hypotheses is the most plausible can potentially be resolved through future
362 intensive sampling of North American Melitaeini butterflies and their *Cotesia* parasitoids.
363 In contrast, Clade D shows an opposite pattern to Clade A: with basal species parasitizing
364 *Melitaea*, followed by a shift to *Euphydryas*. This may reflect a retained ability to use
365 *Euphydryas* inherited from a shared ancestor with Clade A, possibly representing a
366 recolonization event. Species from Clade C are not known to use Melitaeini butterflies but span
367 a wider range of Lepidoptera host taxa. For example, *C. vestalis* parasitizes *Plutella xylostella,*
368 (Linnaeus, 1758) (Yponomeutoidea), a lineage near the base of the Lepidopteran tree (43),
369 while more recently diverged *Cotesia* in this clade parasitize more recently diverged groups
370 such as Pyraloidea (Latreille, 1802) and Noctoidae (Latreille, 1809).
371 Although the relationships within and between cryptic species are better supported in our final
372 phylogeny compared to previous studies (18), comparisons of the tree topologies suggested
373 conflicts between nuclear and mitochondrial reconstructions. The discordance within clades
374 may reflect biological processes such as geneflow between lineages or rapid radiation resulting
375 in incomplete lineage sorting (44,45). The differences between methods and datasets might be
376 due to the non-random absence of mitochondrial data for several taxa in two clades, which can
377 bias phylogenetic inference, especially when relatively few markers are used (46).
378 While we bring an improved version of the *Cotesia* phylogeny, our data still remains too weak to
379 fully evaluate the level of hybridization and introgression between the lineages, or the frequency
380 of host shifts within clades. Additionally, our restricted dataset could have mislead phylogenetic
381 inference especially under incomplete lineage sorting (46). In the future, the use of additional
382 genetic material, specimens from other species, or a wider geographic range will be needed to
383 confidently evaluate the extent of the true evolutionary history of these events.
384 Such increased sampling would also allow for explicit research on the contributions of
385 geography, ecology, and host associations in the diversification of *Cotesia*. For example, other
386 parasitoid lineages show patterns of latitudinal diversity gradients and geographic structuring
387 (47) and similarities might be revealed in *Cotesia*. Additionally, the butterfly host ecology could
388 be explored, which has been observed in parasitoids of leaf miners that often radiate to
389 unrelated hosts that share the same host plant (Leppänen et al., 2012). Finally, studying host
390 associations and range may also influence *Cotesia* diversity through mechanisms such as host
391 fidelity. Whereby many parasitoid wasp females preferentially oviposit on the same host species
392 from which they emerged, reinforcing reproductive barriers and potentially promoting speciation
393 (Forbes et al., 2017; Hood et al., 2015; Kimura and Novković, 2015). Network based
394 approaches provide promising tools to study host-parasitoid coevolution (Braga et al., 2018),
395 testing hypotheses such as adaptive radiation (Ehrlich and Raven, 1964), or host use
396 oscillations (Janz and Nylin, 2008). Understanding host repertoire has important implications for
397 conservation, as species with narrow fundamental host ranges face greater extinction risks, and
398 identifying such species can help prioritize conservation efforts (Farrell et al., 2021).

## 399 Conclusion

400 Out study provides a robust phylogenetic framework for *Cotesia* parasitizing Melitaeini
401 butterflies, clarifying uncertainties in their evolutionary relationships. The results show that the
402 species attacking Melitaeini are paraphyletic, grouping in three of the four major clades,
403 indicating independent host shifts. Although our sampling does not allow for inference of which
404 lineage first adapted to Melitaeini hosts, the confirmation of distinct cryptic species in *C.*
405 *acuminata agg* and *C. melitaearum agg.* highlights the extent of hidden diversity in this system.
406 Together, these findings establish a foundation for future taxonomic revisions, comparative
407 genomic studies, and exploration of the ecological barriers that initiated and maintain their
408 reproductive isolation.

409

## 410 Data Availability

411 NCBI accession numbers for all markers and specimens will be available upon publication
412 (accession numbers have been created for all sequences, cited in the manuscript). The authors
413 are happy to share the sequence files with reviewers upon request.

## 414 Acknowledgements

415 Thanks to the members of the ISEE lab and life-history evolution group at the University of
416 Helsinki for the many discussions and input. Thank you to Dr. Victoria Twort for her input on the
417 manuscript. Thanks to everyone involved in the sample collection, including Constantí
418 Stefanescu and his team in Spain, Yannick Chittaro in Switzerland, and in Finland Suvi Ikonen
419 and Krista Raveala, along with those involved in the Åland butterfly survey. 420

##### 421 References

422 1.	Kjer KM, Simon C, Yavorskaya M, Beutel RG. Progress, pitfalls and parallel universes: a
423 history of insect phylogenetics. J R Soc Interface. 2016 Aug;13(121):20160363.
424 2.	Bickford D, Lohman DJ, Sodhi NS, Ng PKL, Meier R, Winker K, et al. Cryptic species as
425 a window on diversity and conservation. Trends Ecol Evol. 2007 Mar;22(3):148–55.
426 3.	Kon T, Yoshino T, Mukai T, Nishida M. DNA sequences identify numerous cryptic species
427 of the vertebrate: A lesson from the gobioid fish *Schindleria* . Mol Phylogenet Evol. 2007
428 Jul;44(1):53–62.
429 4.	Shin CP, Allmon WD. How we study cryptic species and their biological implications: A
430 case study from marine shelled gastropods. Ecol Evol. 2023 Sep 5;13(9).
431 5.	Grundt HH, Kjølner S, Borgen L, Rieseberg LH, Brochmann C. High biological species
432 diversity in the arctic flora. Proceedings of the National Academy of Sciences. 2006 Jan
433 24;103(4):972–5.
434 6.	Hebert PDN, Penton EH, Burns JM, Janzen DH, Hallwachs W. Ten species in one: DNA
435 barcoding reveals cryptic species in the neotropical skipper butterfly *Astraptes fulgerator*.
436 Proceedings of the National Academy of Sciences. 2004 Oct 12;101(41):14812–7.
437 7.	Li X, Wiens JJ. Estimating global biodiversity: The role of cryptic insect species. Syst Biol.

438	2023 Jun 16;72(2):391–403.

439 8.	Koh LP, Dunn RR, Sodhi NS, Colwell RK, Proctor HC, Smith VS. Species coextinctions
440 and the biodiversity crisis. Science (1979). 2004 Sep 10;305(5690):1632–4.
441 9.	Forbes AA, Bagley RK, Beer MA, Hippee AC, Widmayer HA. Quantifying the
442 unquantifiable: Why Hymenoptera, not Coleoptera, is the most speciose animal order.
443 Vol. 18, BMC Ecology. BioMed Central Ltd.; 2018.
444 10.	Whitfield JB, Austin AD, Fernandez-Triana JL. Systematics, biology, and evolution of
445 Microgastrine parasitoid wasps. Annu Rev Entomol. 2018 Jan 7;63(1):389–406.
446 11.	Cornell H V., Hawkins BA. Patterns of parasitoid accumulation on introduced herbivores.
447 In: Hawkins BA, Sheehan W, editors. Parasitoid Community Ecology. Online. Oxford
448 University Press; 1994. p. 77–89.
449 12.	Lei GC, Hanski I. Metapopulation structure of *Cotesia melitaearum*, a specialist parasitoid
450 of the butterfly *Melitaea cinxia*. Oikos. 1997 Feb;78(1):91.
451 13.	Li X, Jia JJ, An JL, Meng FX, Liu TX, Zhang SZ. Effect of *Cotesia ruficrus* parasitization
452 on diversity and community composition of intestinal bacteria in *Spodoptera frugiperda*.
453 Insects. 2024 Jul 27;15(8):570.
454 14.	Quicke DLJ. The Braconid and Ichneumonid Parasitoid Wasps. Wiley; 2014. 341–449 p.
455 15.	Fernandez-Triana J, Shaw MR, Boudreault C, Beaudin M, Broad GR. Annotated and
456 illustrated world checklist of Microgastrinae parasitoid wasps (Hymenoptera, Braconidae).
457 Zookeys. 2020 Mar 23;920:1–1089.
458 16.	Mason WRM. The polyphyletic nature of *Apanteles Foerster* (Hymenoptera: Braconidae):
459 a phylogeny and reclassification of Microgastrinae. Memoirs of the Entomological Society
460 of Canada. 1981 May 31;113(S115):1–147.
461 17.	Kankare M, Stefanescu C, Van Nouhuys S, Shaw MR. Host specialization by *Cotesia*
462 wasps (Hymenoptera: Braconidae) parasitizing species-rich Melitaeini (Lepidoptera:
463 Nymphalidae) communities in north-eastern Spain. Biological Journal of the Linnean
464 Society. 2005 Sep;86(1):45–65.
465 18.	Kankare M, Shaw MR. Molecular phylogeny of *Cotesia* Cameron, 1891 (Insecta:
466 Hymenoptera: Braconidae: Microgastrinae) parasitoids associated with Melitaeini
467 butterflies (Insecta: Lepidoptera: Nymphalidae: Melitaeini). Mol Phylogenet Evol. 2004
468 Jul;32(1):207–20.
469 19.	Wahlberg , Niklas, Kullberg J, Hanski I. Natural history of some Siberian melitaeine
470 butterfly species (Nymphalidae: Melitaeini) and their parasitoids. Entomol Fenn. 2001 Jun
471 1;12(2).
472 20.	Kankare M, Van Nouhuys S, Hanski I. Genetic divergence among host-specific cryptic
473 species in *Cotesia melitaearum Aggregate* (Hymenoptera: Braconidae), parasitoids of
474 checkerspot butterflies. Ann Entomol Soc Am. 2005 May;98(3):382–94.
475 21.	Valerio F, Martel C, Stefanescu C, Duplouy A. *Wolbachia* strain diversity in a complex
476 group of sympatric cryptic parasitoid species. 2023; Available from:
477 https://doi.org/10.21203/rs.3.rs-3649450/v1
478 22.	Schach J. Molecular Phylogeny of *Cotesia* Wasps Parasitizing Melitaeini Butterflies and
479 the Possible Role of *Wolbachia* Endosymbionts [Master’s thesis]. [Helsinki]: University of
480 Helsinki; 2024.
481 23.	Miles CI, Chen WP, Adamo SA, Kester KM, Miller DW. *Manduca sexta* caterpillars
482 parasitized by the wasp *Cotesia congregata* stop chewing despite an intact motor system.
483 Journal of Experimental Biology. 2023 Aug 15;226(16).
484 24.	Blažytė-Čereškienė L, Aleknavičius D, Apšegaitė V, Būda V. Response of parasitic wasp
485 *Cotesia glomerata* l. (hymenoptera: braconidae) to cabbage plants of two varieties:
486 olfactory spectra of males and females. J Econ Entomol. 2022 Oct 12;115(5):1464–71.
487 25.	Ye X, Xiong S, Teng Z, Yang Y, Wang J, Yu K, et al. Retraction: Amino acid synthesis
488 loss in parasitoid wasps and other hymenopterans. Elife. 2020 Dec 1;9.
489 26.	Gauthier J, Boulain H, van Vugt JJFA, Baudry L, Persyn E, Aury JM, et al. Chromosomal
490 scale assembly of parasitic wasp genome reveals symbiotic virus colonization. Commun
491 Biol. 2021 Jan 22;4(1):104.
492 27.	Pinto BJ, Weis JJ, Gamble T, Ode PJ, Paul R, Zaspel JM. A chromosome-level genome
493 assembly of the parasitoid wasp, *Cotesia glomerata* (Hymenoptera: Braconidae). Journal
494 of Heredity. 2021 Nov 1;112(6):558–64.
495 28.	Muller H, Chebbi MA, Bouzar C, Périquet G, Fortuna T, Calatayud PA, et al. Genome-
496 wide patterns of bracovirus chromosomal integration into multiple host tissues during
497 parasitism. J Virol. 2021 Oct 27;95(22).
498 29.	Burke GR, Strand MR. Deep sequencing identifies viral and wasp genes with potential
499 roles in replication of *Microplitis demolitor* bracovirus. J Virol. 2012 Mar 15;86(6):3293–
500 306.
501 30.	Ye J, Coulouris G, Zaretskaya I, Cutcutache I, Rozen S, Madden TL. Primer-BLAST: a
502 tool to design target-specific primers for polymerase chain reaction. BMC Bioinformatics.
503 2012 Jun 18;13:134.
504 31.	Manni M, Berkeley MR, Seppey M, Zdobnov EM. BUSCO: Assessing genomic data
505 quality and beyond. Curr Protoc. 2021 Dec 22;1(12).
506 32.	Edgar RC. MUSCLE: multiple sequence alignment with high accuracy and high
507 throughput. Nucleic Acids Res. 2004 Mar 8;32(5):1792–7.
508 33.	Larsson A. AliView: a fast and lightweight alignment viewer and editor for large datasets.

509	Bioinformatics. 2014 Nov 15;30(22):3276–8.

510 34.	Minh BQ, Schmidt HA, Chernomor O, Schrempf D, Woodhams MD, von Haeseler A, et
511 al. IQ-TREE 2: New models and efficient methods for phylogenetic inference in the
512 genomic era. Mol Biol Evol. 2020 May 1;37(5):1530–4.
513 35.	Letunic I, Bork P. Interactive Tree of Life (iTOL) v6: recent updates to the phylogenetic
514 tree display and annotation tool. Nucleic Acids Res. 2024 Jul 5;52(W1):W78–82.
515 36.	Kalyaanamoorthy S, Minh BQ, Wong TKF, von Haeseler A, Jermiin LS. ModelFinder: fast
516 model selection for accurate phylogenetic estimates. Nat Methods. 2017 Jun
517 8;14(6):587–9.
518 37.	Chernomor O, von Haeseler A, Minh BQ. Terrace aware data structure for phylogenomic
519 inference from supermatrices. Syst Biol. 2016 Nov;65(6):997–1008.
520 38.	Hoang DT, Chernomor O, von Haeseler A, Minh BQ, Vinh LS. UFBoot2: Improving the
521 ultrafast bootstrap approximation. Mol Biol Evol. 2018 Feb 1;35(2):518–22.
522 39.	Quicke DLJ. We know too little about parasitoid wasp distributions to draw any
523 conclusions about latitudinal trends in species richness, body size and biology. PLoS
524 One. 2012 Feb 15;7(2).
525 40.	Leppänen SA, Altenhofer E, Liston AD, Nyman T. Ecological versus phylogenetic
526 determinants of trophic associations in a plant–leafminer–parasitoid food web. Evolution
527 (N Y). 2012 Dec;no-no.
528 41.	Long EC, Thomson RC, Shapiro AM. A time-calibrated phylogeny of the butterfly tribe
529 Melitaeini. Mol Phylogenet Evol. 2014 Oct;79:69–81.
530 42.	Moore SD. Regulation of host diapause by an insect parasitoid. Ecol Entomol. 1989 Feb

531	14;14(1):93–8.

532 43.	Kawahara AY, Plotkin D, Espeland M, Meusemann K, Toussaint EFA, Donath A, et al.
533 Phylogenomics reveals the evolutionary timing and pattern of butterflies and moths.
534 Proceedings of the National Academy of Sciences. 2019 Nov 5;116(45):22657–63.
535 44.	Zadra N, Rizzoli A, Rota-Stabelli O. Chronological incongruences between mitochondrial
536 and nuclear phylogenies of *Aedes* mosquitoes. Life. 2021 Feb 25;11(3):181.
537 45.	Maddison WP. Gene Trees in Species Trees. Syst Biol. 1997 Sep 1;46(3):523–36.
538 46.	Xi Z, Liu L, Davis CC. The impact of missing data on species tree estimation. Mol Biol

539	Evol. 2016 Mar;33(3):838–60.

540 47.	Castellanos‐Labarcena J, Steinke D, Adamowicz SJ. Anomalous latitudinal gradients in
541 parasitoid wasp diversity—Hotspots in regions with larger temperature range. Journal of
542 Animal Ecology. 2025 Mar 13;94(3):410–22.
543 48.	Kimura MT, Novković B. Local adaptation and ecological fitting in host use of the
544 *Drosophila* parasitoid *Leptopilina japonica*. Ecol Res. 2015 May 4;30(3):499–505.
545 49.	Hood GR, Forbes AA, Powell THQ, Egan SP, Hamerlinck G, Smith JJ, et al. Sequential
546 divergence and the multiplicative origin of community diversity. Proceedings of the
547 National Academy of Sciences. 2015 Nov 3;112(44).
548 50.	Forbes AA, Devine SN, Hippee AC, Tvedte ES, Ward AKG, Widmayer HA, et al.
549 Revisiting the particular role of host shifts in initiating insect speciation. Evolution (N Y).
550 2017 May 16;71(5):1126–37.
551 51.	Braga MP, Guimarães PR, Wheat CW, Nylin S, Janz N. Unifying host-associated
552 diversification processes using butterfly–plant networks. Nat Commun. 2018 Dec
553 4;9(1):5155.
554 52.	Ehrlich PR, Raven PH. Butterflies and plants: A study in coevolution. Evolution (N Y).

555	1964 Dec;18(4):586–608.

556 53.	Janz N, Nylin S. The oscillation hypothesis of host-plant range and speciation. In: Tilmon
557 K, editor. Specialization, Speciation, and Radiation: The Evolutionary Biology of
558 Herbivorous Insects [Internet]. University of California Press; 2008. p. 203–15. Available
559 from: https://academic.oup.com/california-scholarship-
560 online/book/14621/chapter/168758186
561 54.	Farrell MJ, Park AW, Cressler CE, Dallas T, Huang S, Mideo N, et al. The ghost of hosts
562 past: impacts of host extinction on parasite specificity. Philosophical Transactions of the
563 Royal Society B: Biological Sciences. 2021 Nov 8;376(1837):20200351.

564

Figure 2	[Click here to access/download;Figure;Fig 2.tif](https://www2.cloud.editorialmanager.com/pone/download.aspx?id=43344824&guid=5b0967a2-61af-4cac-a78f-50a511e599f0&scheme=1)[<https://www2.cloud.editorialmanager.com/pone/download.aspx?id=43344824&guid=5b0967a2-61af-4cac-a78f-50a511e599f0&scheme=1>](https://www2.cloud.editorialmanager.com/pone/download.aspx?id=43344824&guid=5b0967a2-61af-4cac-a78f-50a511e599f0&scheme=1)

Figure 3	[Click here to access/download;Figure;Fig 3.tif](https://www2.cloud.editorialmanager.com/pone/download.aspx?id=43344825&guid=28cf2751-66b6-40cd-9071-b658b0deffb6&scheme=1)[<https://www2.cloud.editorialmanager.com/pone/download.aspx?id=43344825&guid=28cf2751-66b6-40cd-9071-b658b0deffb6&scheme=1>](https://www2.cloud.editorialmanager.com/pone/download.aspx?id=43344825&guid=28cf2751-66b6-40cd-9071-b658b0deffb6&scheme=1)

Figure 1	[Click here to access/download;Figure;Fig 1.tif](https://www2.cloud.editorialmanager.com/pone/download.aspx?id=43344826&guid=1008a477-0613-4542-b7ea-3723bbc58021&scheme=1)[<https://www2.cloud.editorialmanager.com/pone/download.aspx?id=43344826&guid=1008a477-0613-4542-b7ea-3723bbc58021&scheme=1>](https://www2.cloud.editorialmanager.com/pone/download.aspx?id=43344826&guid=1008a477-0613-4542-b7ea-3723bbc58021&scheme=1)

Supporting Information

### [Click here to access/download](https://www2.cloud.editorialmanager.com/pone/download.aspx?id=43342730&guid=89b31993-49c3-4efd-ae3b-6e4e9bd487b2&scheme=1)

[**Supporting Information** SCHACH_Supplementary material Cotesia Phylogeny_6-1-2026.docx](https://www2.cloud.editorialmanager.com/pone/download.aspx?id=43342730&guid=89b31993-49c3-4efd-ae3b-6e4e9bd487b2&scheme=1)
