# Supplementary material for: Progress in the molecular phylogeny of Cotesia acuminata and C. melitaearum cryptic species complexes
Source: PLoS One. 2026 May 27;21(5):e0348973. doi: 10.1371/journal.pone.0348973 (PMC13215553; doi:10.1371/journal.pone.0348973)
Supplement: Attachment — Submitted filename: Response to Reviewers.docx [file pone.0348973.s021.docx]

**Response to reviewers**

PONE-D-26-02522

Progress in the molecular phylogeny of Cotesiaacuminata and C. melitaearum cryptic species complexes

PLOS One

Dear Dr. Schach,

Thank you for submitting your manuscript to PLOS ONE. After careful consideration, we feel that it has merit but does not fully meet PLOS ONE’s publication criteria as it currently stands. Therefore, we invite you to submit a revised version of the manuscript that addresses the points raised during the review process.

We look forward to receiving your revised manuscript.

Kind regards,

Petr Heneberg
Academic Editor
PLOS One

**Response:** We thank the Editor for taking the time to overview the review of this manuscript. We have now replied to all comments and made changes when appropriate. Please find below the point by point response to all those comments and suggestions. We hope that the new version of this manuscript will now be found suitable for publication in *PLoS ONE.*

Journal Requirements:

When submitting your revision, we need you to address these additional requirements.

1. Please ensure that your manuscript meets PLOS ONE's style requirements, including those for file naming.

2. Please note that PLOS One has specific guidelines on code sharing for submissions in which author-generated code underpins the findings in the manuscript. In these cases, we expect all author-generated code to be made available without restrictions upon publication of the work. Please review our guidelines at <https://journals.plos.org/plosone/s/materials-and-software-sharing#loc-sharing-code> and ensure that your code is shared in a way that follows best practice and facilitates reproducibility and reuse.

**Response to point 1 and 2:** The style requirements of the manuscript have now been edited accordingly to the PLoS ONE guidelines. No Author generated code underpins the findings of this study.

3. We note that the grant information you provided in the ‘Funding Information’ and ‘Financial Disclosure’ sections do not match. When you resubmit, please ensure that you provide the correct grant numbers for the awards you received for your study in the ‘Funding Information’ section.

**Response:** This has now been corrected.

4. Thank you for stating the following financial disclosure:

“The work was funded by the Research Council of Finland to Dr. Anne Duplouy grant number 328944”

Please state what role the funders took in the study. If the funders had no role, please state: "The funders had no role in study design, data collection and analysis, decision to publish, or preparation of the manuscript." If this statement is not correct you must amend it as needed.

Please include this amended Role of Funder statement in your cover letter; we will change the online submission form on your behalf.

**Response:** The funders had no role, other than financial support in this study. This has now been added to the cover letter

5. When completing the data availability statement of the submission form, you indicated that you will make your data available on acceptance. We strongly recommend all authors decide on a data sharing plan before acceptance, as the process can be lengthy and hold up publication timelines. Please note that, though access restrictions are acceptable now, your entire data will need to be made freely accessible if your manuscript is accepted for publication. This policy applies to all data except where public deposition would breach compliance with the protocol approved by your research ethics board. If you are unable to adhere to our open data policy, please kindly revise your statement to explain your reasoning and we will seek the editor's input on an exemption. Please be assured that, once you have provided your new statement, the assessment of your exemption will not hold up the peer review process.

**Response:** All sequence data in NCBI has now been released, with the exception for the sequence data for the *LW-Rh* gene which still needs to be processed but will be available on May 1^st^ at the latest.

6. Please amend either the title on the online submission form (via Edit Submission) or the title in the manuscript so that they are identical.

**Response:** This has now been corrected

7. Please include captions for your Supporting Information files at the end of your manuscript, and update any in-text citations to match accordingly. Please see our Supporting Information guidelines for more information: [http://journals.plos.org/plosone/s/supporting-information.](http://journals.plos.org/plosone/s/supporting-information.%E2%80%8B)

**Response:** This has been completed

8. If the reviewer comments include a recommendation to cite specific previously published works, please review and evaluate these publications to determine whether they are relevant and should be cited. There is no requirement to cite these works unless the editor has indicated otherwise.

**Response:**  One article was suggested by the reviewers. The study was relevant to our work. Thus, we have decided to include it.

**5. Review Comments to the Author**

**Reviewer #1:** The paper presents a multi-gene phylogeny for braconid wasps in genus Cotesia with improved resolution. It is suitable for publication in Plos One after a minor revision.

In addition to the changes made in the attached word document, I have the following five main concerns.

**Response:** We thank the reviewer #1 for their assessment of our work. We have now edited the text according to the reviewers’ suggestions (please see the track changes manuscript), and a point-by-point response to each comment can be found below.

1) the abbreviation agg. should not be italicized, as it is not part of the scientific name of the species. The paper that the authors cite for the use of this term (Kankare et al 2005) also does not italicize the term.
**Response:**  All instances of the abbreviation agg have been changed to non-italicised.

2) Table 1 does not include GenBank numbers for each individual analysed, and it is unclear which gene was sequenced for which sample. From Table 1 it can be inferred that the authors had a total of about 105 individuals (plus some from NCBI). A new supplementary table is needed with a list of these 105 individuals, their collection data and GPS coordinates (even if approximate), followed by columns for each of the 10 genes and the GenBank accessions for each individual clearly indicated.

**Response:**  Following the reviewer’s suggestion, we have added a table to the supplementary material (S1 file). This table includes the accession numbers for each individual and gene, the collection data, and approximate GPS coordinates.

3) The authors mention sympatric occurrence of some of the Cotesia species, however without a map it is difficult to understand the extent of such sympatry. A new separate figure is needed with a map marking the geographic locations of the studied samples. Different symbols and colors should be used for the dots on the map to show: a) samples belonging to the four main clades, and b) whether or not they parasitize Melitaeini.

**Response:** We have created a figure with maps, which include the sampling locations of the specimens. Points are colour coded by clade and the shapes refer to the Lepidoptera host (i.e. Melitaeini or not). Please see Fig 1.

4) It is unclear how the authors decided to separate and highlight their four clades. For example, Clades C and D could be considered a single clade … I suggest that the authors conduct a basic Species Delimitation analysis (such as ASAP or PTP) or something similar, at least on their final phylogeny, to support their clade/species designations and conclusions.

**Response:**  Thanks for the suggestion, we have now ran a bPTP analysis and BPP as recommended by other reviewers. This analysis shows the support for the four clades and cryptic species as previously described in the ms.

5) The authors make no suggestions to improve the systematics of Cotesia. Could the four main clades perhaps be considered subgenera? or species-groups? I suggest the authors to include at least a paragraph about this in their discussion.

**Response:** We now have changed the clades from A-D to represent the species groups instead, additionally, in the discussion we state “Our study questions the need to revise the taxonomy of *Cotesia* to highlight the cryptic diversity, and the possible introduction of new sub-genera by taxonomists.” although we do not feel qualified as taxonomists to describe new sub-genera.

#######################

**Reviewer #2:** The authors undertake a study to investigate phylogenetic relationships among Cotesia species parasitizing the caterpillars of Melitaeini butterflies. They do this using 102 fresh samples of Cotesia combined with and eight species for which data was available on GenBank. Their outgroup consisted of two Microplitis species. They generated data from 10 gene fragments (8 nuclear, 2 mitochondrial) for their taxa and analyzed them in a concatenated framework and by individual gene using maximum likelihood.

The authors show the Cotesia associated with Meliteaini are non-monophyletic and the existence of multiple forms within the currently recognized species C. acuminata and C. melitaerum.

**Response:** We thank Reviewer #2 for their suggestions. We have taken those into consideration and outline the changes in our responses below.

My main critique of the study is the limited approach to phylogenetic analysis and the unsupported statements regarding the production of a “robust” phylogenetic framework.

The authors only use a single analytical approach to phylogenetic inference – maximum likelihood (ML) as implemented in IQ-Tree. While the authors do investigate the influence of combining mitochondrial genes with nuclear genes, and the results of individual gene analyses, these can merely identify contention within the data themselves rather than the effects of different analytical approaches. Most modern phylogenetic studies at least incorporate some form of coalescent approach in order to account for gene tree/species tree discordance and incomplete lineage sorting directly. The authors identify the presence of gene tree discordance through analysis of their individual loci. However, they do not produce a coalescent-based tree to help resolve this. A common software package for this is ASTRAL (<https://github.com/smirarab/ASTRAL>) which makes use of summary coalescence – analyzing a priori produced gene trees to infer a species tree. The authors have already done most the work producing individual ML trees and would simply have to provide these to ASTRAL to produce a species tree. Additionally, given the highly tractable size of their data, they could also perform Bayesian inference (BI) using MrBayes (<https://nbisweden.github.io/MrBayes/>) or Beast (<https://www.beast2.org/tutorials/>).

A highly robust phylogenetic framework would be obtained from reciprocal illumination of results generated using different analytical approaches such as ML, BI, and ASTRAL. If these three fundamentally different approaches produce similar trees, then the data and inferred evolutionary history is robust to analytical approach.

Finally, the results the authors did obtain do not provide robust resolution across their datasets. An ultrafast bootstrap value of ≥95 is considered strong support (see reference 38), anything below this is not strongly supported. Figure 1, Clade (B(C+D)) shows low bootstrap support 77 (the nest node is illegible), Figure 2, the same Clade (B(C+D)) has similarly low bootstrap support – 82, then 87. Finally, figure 3, the analysis of mitochondrial genes alone recovers a different topology where B moves out and A moves in to clade. Bootstrap value is notably missing for this node in Figure 3. Combined these suggest that both within analysis support for much of their recovered topology is not strong, and the same topologies are not recovered across all datasets. So, while the recovery of species-group clades is consistent, relationship among said clades and thus the backbone of the phylogeny is not robust.

I recommend the authors include additional analytical approaches, at the minimum ASTRAL. Ideally, BI and ASTRAL.

**Response:** Thank you for the suggestions. We have added an analysis using ASTRAL to our study. We have edited the method, results and discussion sections to include this work (see methods (pages 13-14) and results (page 20)). In brief, ASTRAL recovered the same major clades as our original ML tree. However, with 10 loci, the signal was too weak to resolve within clade relationships. As similar results were obtained from both analyses, we did not find the need to run a first type of analysis through BI, which is likely to produce the same output based on our dataset. In the future, genomic data will be best analysed using BI, especially in the context of estimating the divergence time between species, clades, or maybe subgenera.

The authors indicate they suspect cryptic species, they could also perform a species delimitation analysis using BPP (<https://github.com/bpp/bpp>) and their mitochondrial data as well as their preferred tree as a guide tree to see how many of these potential cryptic species-level units are supported in a multispecies coalescent framework. Currently they have identified consistent splits in ML trees, but the nature of which of these splits are species-level is unclear, particularly for C. melitaearum which shows less structure than C. acuminata. Species delimitation may support C. melitaerum samples as being part of a single species relative to other taxa in the tree like C. acuminata.

**Response:** Thank you for the suggestion, we have included BPP analysis along with bPTP to test species delimitation, both of which support the presence of cryptic species as described previously (Kankare et al 2005). See results (page 21)

On lines 90 – 92 the authors state they want to resolve basal nodes of Cotesia, suggesting the broader phylogeny is of interest to them. However, based on the rest of the study it really seems the true focus is that discussed in 85 – 87. Therefore, I suggest removing this line as the broader phylogeny of Cotesia was really not a focus.

**Response:** We have removed this line

The authors fail to reference Michel-Salzat & Whitfield 2004 which appears to be an important study on the broader Cotesia phylogeny. This should be referenced.

Michel-Salzat, A. and Whitfield, J.B. (2004), Preliminary evolutionary relationships within the parasitoid wasp genus Cotesia (Hymenoptera: Braconidae: Microgastrinae): combined analysis of four genes. Systematic Entomology, 29: 371-382. <https://doi.org/10.1111/j.0307-6970.2004.00246.x>

**Response:** We thank the reviewer for recommending this article, we have now included the reference in our article.

Finally, a note that the discussion should probably be narrative and not include bullet points. There are also a couple issues with incorrectly formatted references (Line 56 & 83).

**Response:** Thank you for bringing up these mistakes. We have corrected the formatting issues following the instructions from PLOS ONE. Additionally, bullet points are now only present in the results section.

#######################

**Reviewer #3: General**
The submitted manuscript by Schach et al. uses a phylogenetics approach to investigate species complex relationships in a parasitoid wasp group. The analyses use 10 loci (eight nuclear and two mitochondrial) and a concatenated maximum likelihood approach. The results showed that the parasitoid wasp lineages were paraphyletic, resulting from numerous shifts in hosts.
Many recent/current phylogenomic studies utilize Illumina NGS data to isolate hundreds to thousands of loci. While that is common, not every study needs that level of data, especially projects that are long-standing or have a small budget. I am not going to ask for that here, since that seems unrealistic. However, I would say that many of the current analysis approaches used in those study should be at least considered here. Those include a coalescent-based approach such as Astral. Ten loci is not very many, yet there may be enough signal there. The concatenated approach is likely the better option, as shown here, but more work should be done to show concordance/discordance in the data used. Approaches such as PhyParts or gene concordance factors would show how many of the individual genes support different nodes in the concatenated tree. Bootstrap support, which tends to be inflated in phylogenomic data sets, is primarily a metric of confidence in the topology, not so much in the level of support of the data (either genes or sites) of the topology. In addition to bootstrap support, it would be useful to see how site concordance factors support the concatenated topology. [...] Overall, there is nothing wrong with the underlying data. This is a great foundation for a solid manuscript, but some reworking of analyses and revision of the main text is needed before publication. One specific example is the talk of species or cryptic species; a monophyletic group in a phylogenetic tree may not be enough evidence to assign species. There are numerous species delimitation approaches that should be at least considered if some of the states want to be kept.

**Response:** We thank reviewer #3 for taking the time to review this manuscript and for insightful suggestions, which have helped us improve the quality of this manuscript. Thank you also for their encouragements, which have supported the learning and motivation of all co-authors. We have included additional analyses, including gene concordance factors on the concatenated analyses, coalescent based approach with ASTRAL and species delimitation analyses with BPP and bPTP. We hope that these additional analyses will satisfy the curiosity of the reviewers.

I have a lot of specific comments to follow, many of these involve the presentation of the study. There are numerous grammatical or punctuation mistakes throughout. A careful revision is needed. Towards the end of the discussion the formatting of the intext citations completely changes from the rest of the manuscript.

**Response:** Thank you for pointing these out. We have reformatted the reference list and corrected typos highlighted by the reviewers.

I also have many comments involving being explicit. Instead of saying “a few”, say how many. When saying that mito and nuclear loci have been used previously, say which ones. These things will help nonexperts in parasitoid wasp evolution understand the manuscript.

**Response:** We have corrected this to explicitly state which loci have been used.

Line 15: I suggest checking the author guidelines and see if keywords need to be alphabetical

**Response:** There are no particular guidelines for keywords for the PLoS ONE journal, as keywords are optional for this journal. However, we place them in alphabetical order.

Line 21: “…with limited sampling…”; Taxonomic, population, or genetic/genomic sampling?

**Response:** This has been updated to include both taxonomic and genetic sampling

Line 39: As currently written, I don’t think the comma after cryptic species is necessary

**Response:** The comma has been removed

Line 51: In cases like this, isn’t it better to write “Lei and Hanski (12), instead of just the number?

**Response:** This sentence has been changed to: For example, it has been shown that the specialist species *Cotesia melitaearum* (Wilkinson), parasitizes only 10% of *Melitaea cinxia* (Linnaeus) caterpillars but can still cause localized extinctions within the butterfly host metapopulation [12].

Line 58: missing a period at the end of the sentence.

**Response:** A period has been added

Line 59: missing an apostrophe in worlds, since its not multiple worlds

**Response:** An apostrophe added

Line 63-64: This sentence is a bit confusing. Cotesia has 300 named species where was Microgastrinae is 1500-2000, yes? I suggest revising to make it more clear

**Response:** *Cotesia* has 1500-2000 estimated species with over 300 of them being already named. The sentence has been changed to: Within the Braconidae, the subfamily Microgastrinae is the most diverse. For example, the genus *Cotesia* (Cameron) contains an estimated 1500-2000 species worldwide [16] with over 300 of which have been named as species [17].

Line 64: The “the” starting the sentence can be deleted and just start with Cotesia

**Response: “**The” has been removed

Line 66: how many is “few”?

**Response:** We have changed few to two making it more explicit: *Cotesia* wasps usually exhibit narrow host ranges, and closely related species often differ in their host species preferences, attacking one or two closely related lepidopteran host species [18]

& Line 78: how many are “few”? Please be explicit

**Response:** The sentence have been updated to be more specific Currently, the phylogeny of the Catalonian *C. acuminata* complex and *C. melitaearum* complex are based on 12 microsatellite loci, while a wider Eurasian phylogeny of these groups combined two mitochondrial (*COI* and *ND1*) and one nuclear (ITS2) gene sequences [19].

Line 67: should Melitaeini be italicized here? The presence of an authority suggests yes

**Response:** Melitaeini is the tribe, therefore it should not be italicized

Line 70: comma between the two species can be removed. Has C. melitaearum been used previously, it doesn’t have an authority

**Response:** The comma has been removed. ‘*C. melitaearum'* was used in line 52 (line 48 in the revised MS) and has the authority

Line 73: I think this is another case when providing the author names would be helpful; also, I don’t think “indeed” is necessary

Line 74: comma between species and are is not necessary

**Response to line 73 and 74:** The sentence has been updated to: ‘In the complex species community of Melitaeini butterflies occurring in Catalonia in northeastern Spain (Catalan Butterfly Monitoring Scheme, CMBS), 10 local Melitaeini butterfly species were identified as hosts to at least 10 *Cotesia* (cryptic) species [18].’

Line 79-80: which two mito genes? Which nuclear locus? Please be explicit
**Response:** The names of the genes have now been added

Line 82: What values do the author consider “low bootstrap”? Under 70? Under 50?
**Response:** We have updated the analysis to use non-parametric bootstrapping instead of ultra-fast and have clearly stated in the methods that we consider values under 60 to be unsupported.

Line 83: Why are the citations here formatted different than all others?
**Response:** The error came from an issue with the reference manager used for the previous version of the ms. All references have been corrected to match the suggested format of PLOS ONE.

Line 85: The abstract says 22 species. What is the disconnect between 22 and 16?

**Response:** The abstract says 22 species of Cotesia, while this line mentions they are attacking 16 species of butterfly.

Line 86: comma after butterflies is not necessary; how many markers? 10?

**Response:** Thank you. The study includes 10 markers, which we now explicitly state. Additionally, the comma was removed

Line 97: missing a comma between the first two species

**Response:** Comma added

Line 98: Can abbreviate the genus name in the list after the first use, especially when there are over 8 for each genus

**Response:** As suggested by the reviewer, we have edited this section such that the Genus name is abbreviated after the first use.

Line 105: similar to above, would be helpful to write out author names before (20)

**Response:** We changed the sentence to: ‘Following previously introduced convention by Kankare et al [21], we refer to these as *C. acuminata* agg*.* and *C. melitaearum* agg*.,* with individual lineages denoted by species-level designations (e.g. *Sp. A* to *Sp. H*)’

Line 109-110: Is the second half of this sentence with “for the study of Cotesia wasps’ phylogeny and butterfly-host specialization in earlier studies….” Needed

**Response:** This has been removed

Line 112-115: If the numbers are 1-9, they should be written out, such as one species, two species, etc. Cases of N=X can remain numbers

**Response:** This has been changed

Line 116: Should fall be capitalized?

**Response:** No, seasons are not capitalized

Line 121: Is manipulated the best word here?
**Response:** Manipulated was changed to: DNA extraction

Line 139: I would make it clear in the Table legend that the GenBank numbers are for reference genomes

**Response:** Thank you for the suggestion. This was updated accordingly.

Line 149: Technically BLAST is a method not a verb; I suggest revising to “BLAST search” with the appropriate citations for BLAST
**Response:** This sentence has been updated to: We used BLAST search against the respective genomes to extract the sequences of the genes used in the phylogeny [26].

Line 163: should cite the previous studies here, especially since “…primer pairs designed and tested by previous studies…”
**Response:** The references have been added here as well

Line 164: Extra period after Table 2 here. Also, I thought Table 2 was for the genome assemblies used, not phylogenies?

**Response:** The extra period has been added, and this table is now Table 3

Line 176-178: Since above there was clear distinction between mito and nuclear, I would make that clear in the table as well. How is it possible that two loci do not have amplicon size? If they amplified, it should be fairly clear what the size is

**Response:** The amplicon sizes are missing for these two, since the original source did not have them listed. Additionally, after we amplified these regions ourselves, we realized that they are highly variable in size between individuals due to large insertions/deletions (e.g. size range for *ITS2* ≈ 153 to 341bp).

Line 183: What was the DNA concentration? Were they normalized across samples to ensure close to equal inputs?

**Response:** The samples were not normalized to a particular concentration and ranged in concentration from 90-470. This has been updated in the methods section.

Line 187: I would merge 1% gel electrophoresis and 30 minutes at 100 volts into the same sentence

**Response:** These have been combined

Line 208: the comma between species is not needed here

**Response:** The comma has been removed

Line 210: What settings for Muscle were used?
**Response:** We have updated this to add “using default settings”.

Line 211: Version of AliVew? Manually curated how?
**Response:** AliView version has been added, and the sentence was updated to: ‘checked by eye with AliView’

Line 216-218: Were the topologies of these different subsets compared quantitatively, such as comparePhylo in Ape to get the number of shared nodes or splits?

**Response:**  We have decided not to compare them quantitatively, only visually if the major clades were recovered.

Line 221: Why was BIC used for model selection instead of AIC given that likelihood and not Bayesian methods were used?

**Response:** We used BIC as the default used in IQTREE2.

Line 224: With a relatively small data set, why not use 1000 regular bootstraps instead of ultrafast? Why not consider using site concordance factors or gene concordance factors as well, since these metrics give a good idea of support instead of just confidence

**Response:** We have rerun the analysis using non-parametric bootstrapping and added gCF to the analysis as well. Which recovered the same clades.

Line 235: Is there a table that includes gene accession numbers for each locus for each species? How many species only had 1 or 2 genes? Overall level of missingness in the data set?
**Response:** This table has been added to the Supplementary materials as S1 File

Line 243: why include parsimony informative character stats if parsimony wasn’t used?

**Response:** This has been removed.

Line 246: Four clades were mentioned but only three support values were given. What happened to the missing value?

**Response:** The support values have been updated for each clade.

Line 282: statistics on how congruent the two trees were to each other?

**Response:** We did not statistically test how congruent the trees are because the three main trees have differing numbers of taxa/specimens due to missing data.
Line 328: How was discordance characterized? The methods don’t suggest additional analyses for concordance/discordance.

**Response:** Concordance was only characterized by visually comparing the topology.

Line 331-332: Was a coalescent based approach tried, such as Astral, with the individual genes?

**Response:** Analysis with Astral has been added, please see page 13 (methods) and 20 (results)

Line 344: apostrophe isn’t needed here since multiple specimens are talked about, not possessive

**Response:** The apostrophe was removed

Line 345: If species delimitation methods were not employed, even though they are in separate clades, can they truly be called different species?

**Response:** We conducted species delimitation testing with BPP and PTP which support our species assignments

Line 354: The term basal can be highly misleading since phylogenies can rotate. Can this be revised to be more explicit?

& Line 363: again, basal can be misleading

**Response:** we have changed basal to ancestral to hopefully make it more clear

Line 355: What are the dates of divergence?

**Response:** The divergence dates for the hosts have been added (42.68 MYA).

Line 373-374: likely, yes, but there is not any expectation that every locus is going to show the exact same pattern. See Larson et al. 2026 in Evolution, discordance in the phylogenomics age.

**Response:** Thank you for the interesting read, we agree. Exploring the processes behind the discordance will be part of future work using whole genome data.

Line 375: “… differences between methods…” what differences in methods?

**Response:** We have removed this.

Line 380-381: This sentence seems to tear down the authors results. Do they not trust the results at all then?
**Response:** the sentence has been edited to: ‘Although whole genome sequencing approaches might provide more detailed information on hybridization and introgression events, or on lineage sorting in the genus *Cotesia,* the integration of new experimental and field data on host fidelity (as in Kankare et al [18]) and/or reproductive isolation would reveal elements of the ecological barriers that have facilitated the speciation of *Cotesia* wasps.’, Our results are robust, but we only wished to be critical of our sample size.

Line 389: References from here on are not formatted the same as all the others

**Response:** We have fixed all the issues with the references.

#######################

Reviewer #4: Dear authors,

I have reviewed your manuscript entitled "Progress in the molecular phylogeny of Cotesia acuminata and C. melitaearum cryptic species complexes." It is very nice work, although it requires some changes. [...] In general, your study presents a very interesting topic worthy of publication. However, before final acceptance, I would like to see the above-mentioned additions to your manuscript.

I also have quite a few minor comments and edits mentioned directly in the text.
With best regards,
Petr Janšta

**Response:** We would like to thank reviewer #4 for commenting on our ms. We have considered all comments made directly in the PDF, and made changes in the text accordingly (see tracked change document).

First of all, I would recommend changing the title, as the study is not only about Cotesia acuminata and C. melitaearum cryptic species complexes but also other species. I would also suggest removing "Progress in the" and simply using "Molecular phylogeny of..."

**Response:** Thank you for the suggestion, however the main focus of this study was the cryptic complexes and closely related species. The inclusion of non-Melitaeini specialists served to determine the monophyly of the species that do attack Melitaeini butterflies. Furthermore, this study builds on previous works (e.g. Molecular phylogeny of *Cotesia* Cameron, 1891 (Insecta: Hymenoptera: Braconidae: Microgastrinae) parasitoids associated with Melitaeini butterflies (Insecta: Lepidoptera: Nymphalidae: Melitaeini)), thus we will not change the title.

Second—and this is my major comment—you work with many cryptic species within C. acuminata and C. melitaearum, but no rigorous analysis (such as single-locus species delimitation, e.g., bPTP, GMYC, etc., or multilocus species delimitation, e.g., SODA) is presented. I believe that once you distinguish cryptic species, a statistical approach such as delimitation needs to be applied. I would also expect you to calculate genetic distances for particular loci and compare them within this study and with other studies.

**Response:** As requested by several reviewers, We have implemented bPTP along with BPP for species delimitation. We have also conducted additional ASTRAL analysis. The Genetic distances comparison in this group of parasitoids is complicated as this is the first study to have these genes sequenced for many of the species included. Additionally, comparing our data to data from Kankare et al 2025 in a way suggested by the reviewer would not make sense as we have sequenced many of the same specimens than Kankare had sequenced (i.e. for the COI gene for example).

I did not find Figure 1 in the MS. If I just overlooked it, I apologize.

**Response:** It seems the order of the figures was shuffled, and Fig 1 was placed last.
